# Rhythmic syllable-related activity in a songbird motor thalamic nucleus necessary for learned vocalizations

**DOI:** 10.1371/journal.pone.0169568

**Published:** 2017-06-15

**Authors:** Husain H. Danish, Dmitriy Aronov, Michale S. Fee

**Affiliations:** Department of Brain and Cognitive Sciences, McGovern Institute for Brain Research, Massachusetts Institute of Technology, Cambridge, MA, United States of America; Utrecht University, NETHERLANDS

## Abstract

Birdsong is a complex behavior that exhibits hierarchical organization. While the representation of singing behavior and its hierarchical organization has been studied in some detail in avian cortical premotor circuits, our understanding of the role of the thalamus in adult birdsong is incomplete. Using a combination of behavioral and electrophysiological studies, we seek to expand on earlier work showing that the thalamic nucleus Uvaeformis (Uva) is necessary for the production of stereotyped, adult song in zebra finch (*Taeniopygia guttata*). We confirm that complete bilateral lesions of Uva abolish singing in the ‘directed’ social context, but find that in the ‘undirected’ social context, such lesions result in highly variable vocalizations similar to early babbling song in juvenile birds. Recordings of neural activity in Uva reveal strong syllable-related modulation, maximally active prior to syllable onsets and minimally active prior to syllable offsets. Furthermore, both song and Uva activity exhibit a pronounced coherent modulation at 10Hz—a pattern observed in downstream premotor areas in adult and, even more prominently, in juvenile birds. These findings are broadly consistent with the idea that Uva is critical in the sequential activation of behavioral modules in HVC.

## Introduction

Many complex behaviors observed in nature exhibit a behavioral hierarchy, a system in which behaviors can be divided into ‘units’ which themselves can be divided into simpler subunits [1]. This hierarchical organization of behavior leads to key questions regarding the neural implementation of complex motor behaviors: are elements of the hierarchy explicitly represented in neural circuits? And if so, how are behavioral units or subunits represented and initiated? Here, we will examine these questions in the context of learned vocalizations of the songbird.

Birdsong is a hierarchically organized complex behavior. The song of adult zebra finches consists of a stereotyped sequence of 2–8 song syllables, which together form a repeated song motif of about 0.5–1 second duration. This motif may be repeated multiple times to form a bout. In addition, bouts of singing are often preceded by a series of short, soft vocalizations called introductory notes [2]. It is unclear how this behavioral hierarchy is represented in either the vocal premotor or the vocal learning circuits of the avian brain.

**Fig 1:**
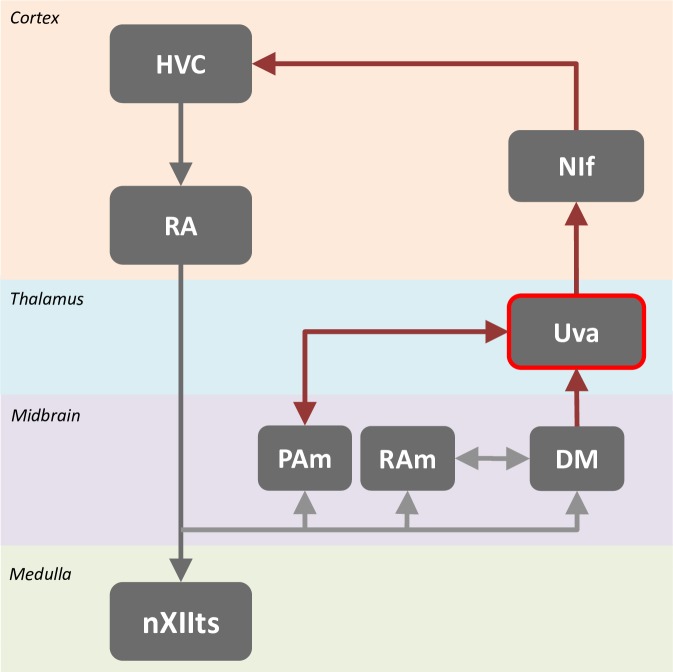
The avian premotor song circuit can be viewed as a combination of a feed-forward premotor pathway (gray arrows) combined with a feedback pathway (brown arrows). HVC (used as proper name); RA (robust nucleus of the arcopallium); nXIIts (tracheosyringeal portion of the hypoglossal nucleus); PAm (nucleus parambigualis); RAm (nucleus retroambigualis); DM (dorsomedial medial nucleus of the intercollicular complex); Uva (nucleus Uvaeformis); NIf (nucleus interface).

The avian vocal network that generates song can be viewed as a combination of both a feed-forward premotor pathway, as well as a feedback pathway (Fig 1). The feedforward pathway includes the nucleus HVC (used as a proper name) [3–5], a premotor nucleus in the avian analog of the mammalian neocortex [6,7]. HVC projects to the robust nucleus of the arcopallium (RA) [3], an avian homologue of layer V primary motor cortex [8,9]. Neurons in HVC that project to RA individually generate a single burst of spikes during song, and as a population generate a sparse, stereotyped sequence of bursts that spans the song motif [10–12]. RA neurons, which individually generate complex sequences of bursts during singing [10,13], provide inputs to motor neurons in the hypoglossal nucleus that innervate muscles of the vocal organ [14]. Neurons in RA additionally project to various brainstem respiratory nuclei and other midbrain motor nuclei [15–19]. Many of these, including the respiratory areas, send a projection back to HVC through the higher-order thalamic nucleus Uvaeformis (Uva) [20,21]. Besides HVC, Uva also projects to two other forebrain nuclei in the song system: NIf and Avalanche (Av) which in turn also project to HVC [22]. In short, Uva forms a key node in a brainstem-thalamocortical loop by which information about the ongoing song vocalization could be transmitted back to the premotor nucleus HVC [23–25].

Prior studies have found that lesions of Uva in adult birds lead to pronounced disruption of song. Carried out in the context of directed (courtship) singing, partial unilateral or bilateral Uva lesions have been shown to disrupt the stereotyped order of syllables within a motif [26]. More complete lesions of Uva have an even more pronounced effect on directed song, transforming the normally stereotyped sequences of syllables into long trains of repeated introductory notes that never transition to an ordered series of song syllables [27].

Several models of Uva function have been proposed based on anatomical connectivity and the dramatic effects of Uva lesion on song. Because Uva is part of a bilaterally connected ascending pathway, it has been suggested that Uva may synchronize activity in the two hemispheres of the telencephalon during singing [24,28–30]. It has also been hypothesized that Uva may play a role in integrating motor corollary discharge signals from RA with information regarding the animal’s respiratory state during singing [24].

Other models of Uva function have been proposed in relation to its possible role in activating sparse bursting in HVC. In one model of Uva/HVC interaction, bursts of activity in HVC projection neurons occur only at discrete times in the song corresponding to syllable onsets and offsets, abrupt transitions in acoustic structure, and extrema of control parameters [31,32]. It has been hypothesized that thalamic input from Uva directly drives activity in HVC at these discrete times [32].

In other models of Uva/HVC interaction, the sparse bursts of individual HVC projection neurons form a continuous sequence that as a population drives downstream motor patterns at every moment [13,33–35]. In one version of this model, the sequential bursting in HVC may result from propagation of activity through synaptically connected chains of neurons within HVC [12,34–38]. For example, each different syllable could be encoded by a different synaptic chain in HVC. Uva could link these chains in HVC such that the end of one syllable chain activates the beginning of the next syllable chain through the Uva feedback loop [12,39,40]. This model predicts that Uva should generate a burst of activity prior to each song syllable [39]. An alternative model suggests that sequential activity in HVC results not from chains in HVC, but from bursts propagating rapidly around the loop from HVC to the midbrain to Uva and back to HVC [25]. This latter model predicts that activity in Uva should continue uninterrupted through song.

An earlier electrophysiological study of Uva reported premotor bursts immediately prior to calls and introductory notes, as well as increased activity during song motifs [26]. This study also reported bursts of activity locked to the offsets of song motifs and song bouts, but did not report syllable-related modulation. One potential difficulty is that this earlier study did not distinguish between the HVC-projecting core of Uva and the non-HVC projecting shell surrounding Uva. Here we have taken advantage of the technique of antidromic stimulation to specifically target the HVC-projecting core of Uva. This approach is advantageous because Uva is a very small nucleus (~250μm dia) located 5.2mm below the dorsal brain surface and thus is difficult to target.

Here we use a combination of lesions and electrophysiological recordings to address the role of Uva in adult song. As previously reported, we find that complete bilateral Uva lesions abolish stereotyped directed adult song. However, we also find that during undirected singing, such lesions result in subsong-like vocalizations that have no distinct identifiable syllables and have a broad, nearly exponential distribution of syllable durations. By specifically targeting and recording from the HVC-projecting core of Uva in singing birds, we find that the HVC-projecting portion of Uva exhibits elevated multiunit activity during song with a distinct pattern of activation prior to syllable onsets and dips prior to syllable offsets. These modulations are are also coherent with a pronounced 10Hz rhythmicity in song structure. Altogether, our findings suggest that activity in Uva is strongly related to syllable onsets and offsets, and in particular with the periodic component of these events. We find no evidence for a specific representation in Uva of other aspects of the song behavioral hierarchy such as song motifs or song bouts.

## Results

### Uva is necessary for adult stereotyped song

**Fig 2:**
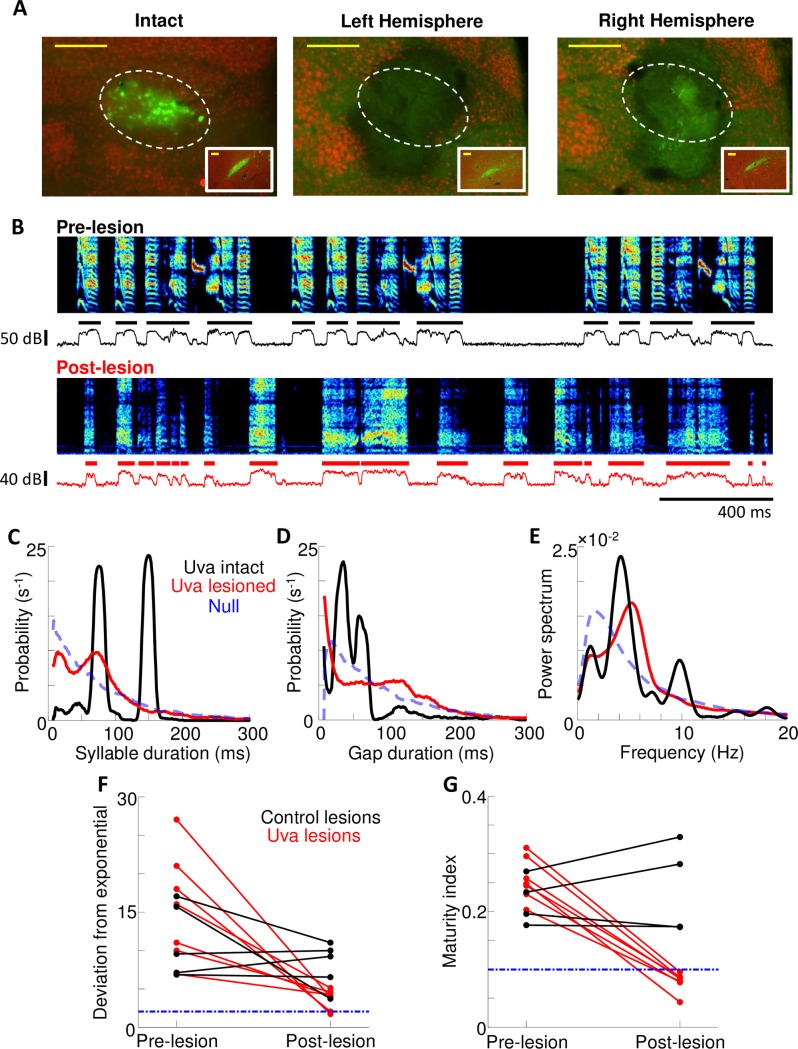
Uva lesions abolish song stereotypy. **(A)** (left) Retrograde tracers from HVC (dextran-conjugated Alexa Fluor 488) were used to distinguish Uva-HVC projectors from surrounding thalamic neurons (Neu-N, red) in an intact bird (yellow scale bar = 200μm). (middle, right) Absence of retrograde tracer or Neu-N stain reveals bilateral elimination of Uva following passing of current into the center of each Uva. Dotted-line marks the border of Uva. (*Inset)*retrograde labeling from HVC in intact NIf demonstrate successful retrograde tracing (scale bar = 200μm) **(B)** (top) Prelesion song spectrogram of an adult bird (>90dph). Bottom trace is the song amplitude and the black segments indicate individual syllables. (Bottom) song spectrogram of the same bird taken from the first day of singing after bilateral Uva lesions. Note the loss of song stereotypy in the duration of syllables and gaps between syllables, as well as the acoustic features of the song. **(C)** and **(D)** Distribution of syllable and gap durations, respectively, before (black trace) and after(red trace) bilateral Uva lesion. The null distribution for syllables and gaps is represented by an exponential or unimodal distribution, respectively (dashed blue trace). **(E)** normalized power spectra of the song amplitudes before and after lesion. Note that the song remains rhythmic after Uva lesions, with a peak in the power spectrum between 4-8Hz. Null power spectrum distribution (dotted blue) was generated from an exponential distribution of syllable durations and a unimodal distribution of gap durations. **(F)** Change in goodness of fit–a metric that quantifies how similar the syllable duration distribution is to that of subsong—in control lesion(black trace) and Uva lesioned birds (red trace). Dashed blue line represents the cutoff for subsong. **(G)** Change in maturity index in control versus Uva lesioned birds. Dashed blue line represents the cutoff for subsong.

To examine the role of Uva in song production, we performed bilateral lesions of Uva in adult male birds (n = 7). Electrolytic lesions were carried out after mapping Uva by antidromic stimulation from HVC, and were confirmed by subsequent histology that included retrograde tracing from HVC (Fig 2A). Only birds with lesions greater than 90% were considered for further analysis. Pre-lesion and post-lesion vocalizations were recorded both in social isolation (undirected song) and during the presentation of a female bird (directed song). When presented with a female, lesioned birds demonstrated typical courtship behaviors, including approach and bill wiping [2]. However, consistent with previous reports [26,27], lesioned birds failed to sing and only produced sporadic short sounds, acoustically similar to introductory notes but without their characteristic rhythmicity (S1 Video).

While Uva lesions completely abolished directed singing, lesioned birds in social isolation sang at rates similar as intact birds in social isolation. However, these vocalizations exhibited highly abnormal acoustic and temporal structure (Fig 2B). Visual inspection of song spectrograms reveal no apparent shared elements between pre- and post-lesion song. Furthermore, post-lesion song have no identifiable motif, and do not appear to contain syllables of reliable acoustic or temporal structure. Consistent with visual inspection of the pre- and post-lesion song, Uva lesions cause a significant decrease in a measure of song repeatability (maturity index; *M*_*pre*_ = 0.26±0.03, *M*_*post*_ = 0.08±0.02; p<0.025, Wilcoxon signed rank test, n = 7 lesioned birds, ±SD, see Methods) (Fig 2G).

Uva lesions also have a characteristic effect on the duration of syllables and the gaps between syllables during undirected song. Intact adult song contains several distinct syllables that form multiple narrow peaks in the distributions of syllable durations. In contrast, the syllable duration distributions of Uva-lesioned birds resemble the broad exponential distribution previously described for subsong birds (Fig 2C) [41]. The extent to which these distributions deviated from exponential was quantified using Lillifors statistic [42] (see Methods). Post-lesion syllable duration distributions are significantly closer to exponential (Γ_post_ = 3.7±1.2) than are pre-lesion songs (Γ_pre_ = 16±6) (p<0.025, Wilcoxon signed rank test, n = 7 lesioned birds) (Fig 2F), but do not meet the strictest criterion for subsong (Γ < 2). Indeed, in the majority of Uva-lesioned birds (n = 6/7), a small peak is seen in the syllable distribution at 50-100ms that is not observed in either subsong or HVC lesioned song [43].

Uva lesions also had a dramatic effect on the silent intervals (gaps) between syllables. Intact adult song contains gaps of discrete durations, forming multiple narrow peaks in the gap duration distribution. Following Uva lesions, however, gap durations become more broadly distributed, with an increased incidence of long and short gaps, similar to the distribution of gap durations previously reported in subsong birds [41,44].

Next, we analyzed song rhythmicity, computed as the power spectrum of sound amplitude during singing. It has been shown that, during development, as vocalizations become more stereotyped zebra finch song acquires more rhythmic temporal structure [43,45–48]. Given the loss of stereotypy in the song of Uva-lesioned birds, we expected the song to have low rhythmicity, with the power spectrum of post-lesion song exhibiting an exponential distribution similar to that seen in subsong birds. Indeed, the increase in variability in both syllable and gap durations following Uva lesions is accompanied by a significant decrease in rhythmicity of song temporal structure in post-lesion birds (Fig 2E; *R*_*pre*_ = 3.30±1.1, *R*_*post*_ = 1.7±0.3; p<0.025 Wilcoxon signed rank test, n = 7 lesioned birds, see Methods). However, unlike subsong birds that had no significant rhythmicity, 6 of 7 Uva-lesioned birds exhibited a significant peak in the power spectrum between 3-8Hz.

We considered the possibility that the effect of lesions targeted to Uva was due to unintended damage to surrounding thalamic tissue, which in our dataset was largely restricted to regions dorsal or ventral to Uva. We directly tested this possibility in four control birds for which the Uva-lesion protocol was carried out exactly as for experimental birds, but the lesion was targeted either 250μm more dorsal (n = 2 birds) or 200μm more ventral (n = 2 birds). We found that, in all cases, these control lesions had no effect on song structure as assessed in song spectrograms, nor did they have a significant effect on syllable or gap duration distributions (p>0.50 for all measurements, n = 4 birds), on maturity index (p>0.50), or song rhythm spectrum (p>0.50) (S1 Fig).

### Song-related activity in Uva

**Fig 3:**
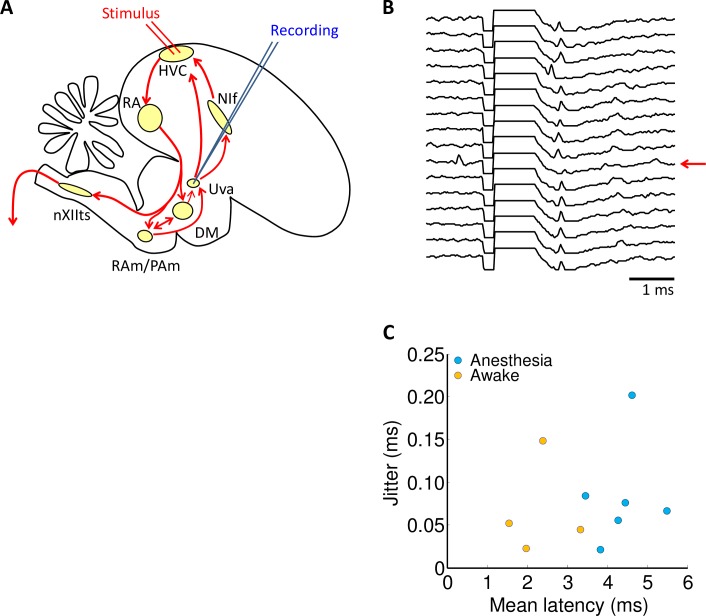
Multi-unit recordings in Uva during singing. **(A)** Simplified schematic view of the oscine song control system. Multiunit recordings were made in HVC-projecting core of Uva, which was antidromically identified by electrical stimulation in HVC. **(B)** Antidromic activation of neurons in Uva. Traces shows the response in Uva across sequential stimulations. Red arrow indicates a trial during which a spontaneous spike occurred, preventing in antidromic response in Uva. **(C)** Latency and jitter of antidromic responses in awake and anesthetized birds.

Our lesion results demonstrate that Uva is necessary for stereotyped, adult song. To elucidate the nature of Uva activity during singing, we recorded from Uva in freely behaving adult zebra finches (n = 5). We targeted recording electrodes to the HVC-projecting core of Uva using antidromic activation from HVC (Fig 3A). Single-unit recordings of antidromically-identified neurons projecting to HVC could be obtained in anesthetized or awake non-singing birds (Fig 3B), but only multiunit signals could be recorded during singing. This was likely due to a large increase in Uva firing rates during singing that prevented single-unit isolation. Antidromic responses had short latencies (1.5-5ms) with a small jitter (<100μs), and could be elicited by stimulation intensity of 70–300μA (Fig 3C). Single-unit recordings of HVC-projecting Uva neurons during non-singing revealed regular spontaneous spiking at 20-50Hz (n = 4 neurons, n = 2 birds). Single-units recorded just dorsal or ventral to HVC-projecting core of Uva appeared to exhibit much lower rates of spontaneous spiking (n = 5 neurons, <10Hz).

**Fig 4:**
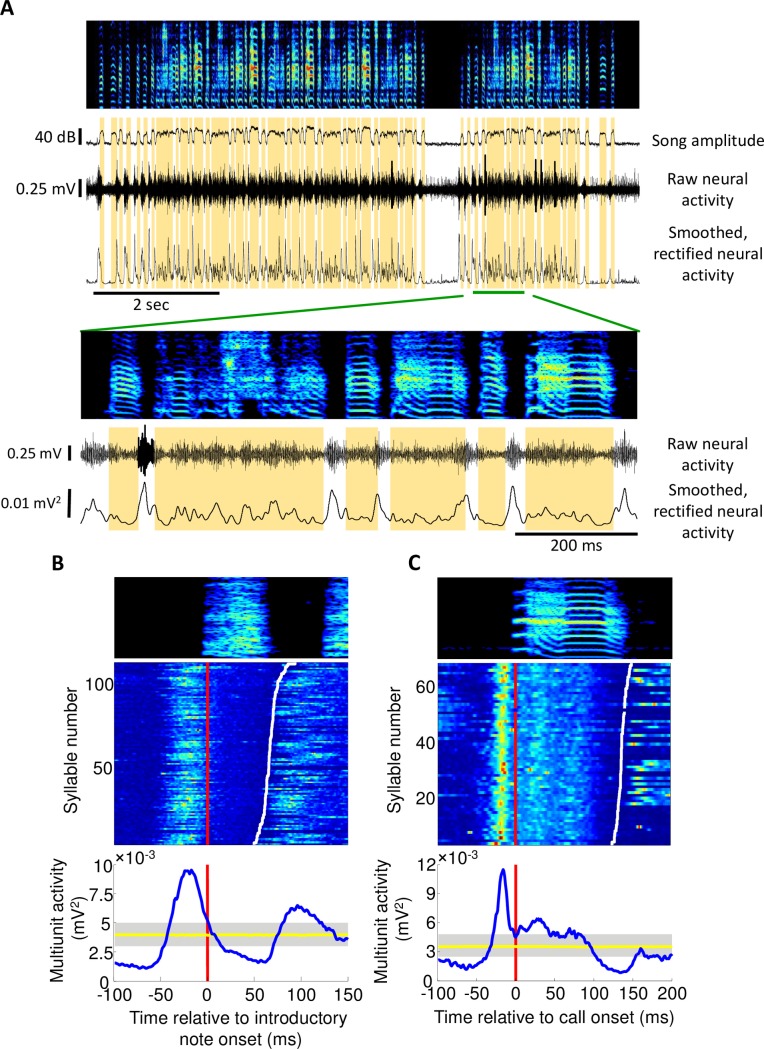
Premotor activity in Uva. **(A)** A trace of neural activity in Uva during a single bout. Song spectrogram (top) of an adult bird (>90dph) followed by a song amplitude trace. Immediately below that is the raw neural activity followed by a smoothed and rectified neural trace. Orange bars mark out individual syllables. Detailed examination of Uva activity during a song motif reveals peaks in activity prior to syllable onsets. The last syllable in the song bout is followed by a period of depressed neural activity in Uva lasting for approximately 200ms. **(B)** Uva exhibits activity prior to onset of introductory notes. At the top is a spectrogram of a single, example introductory note. Raster represents the power of neural activity during each rendition of an introductory note. Red line marks introductory note onset and white line marks introductory note offset. Below is a note onset aligned multiunit trace averaged across all renditions. Also shown is the baseline activity during vocalization determined from random shuffling of multiunit activity (yellow; shading indicates 95% confidence interval for maxima and minima anywhere in this window). **(C)** Uva activity during distance calls. Same as (**B**) but with distance calls instead of introductory notes. Note the peak in activity prior to call onset.

**Fig 5:**
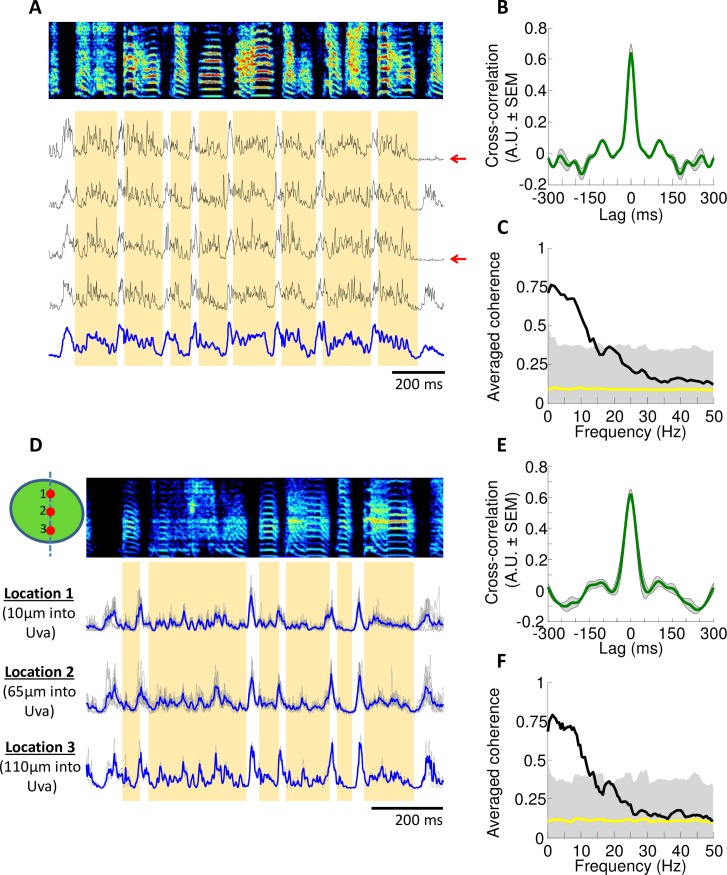
Uva exhibits consistent activity across multiple song renditions at multiple recording sites. **(A)** Activity in Uva is consistent across multiple bouts. From top to bottom, spectrogram of a single motif, multiple traces of time-warped, smoothed and rectified multiunit activity and an average trace in blue. Red arrows indicate motifs that occur at an end of a rendition. Orange bars mark out each syllable. **(B)** Cross-correlation across multiple renditions (shaded region indicating SEM) **(C)** Coherence across multiple renditions (shaded region indicating the 95% percentile of the null distribution corrected for multiple testing). **(D)** Activity in Uva is consistent across different recording sites. An average trace of time-warped multiunit activity at each recording site is shown in blue. Individual traces are shown in gray. Diagram in upper left-hand corner represents the relative position of each recording site within Uva. **(E)** Cross-correlation and (**F**) coherency across multiple recording sites.

Multiunit activity in Uva exhibited strong modulation related to vocalizations. The multiunit signal was quantified by first rectifying and then smoothing the raw microelectrode signal (see Methods). As previously reported [26], Uva activity increases sharply immediately prior to the onset of distance calls (Fig 4C; latency from baseline = 30±7ms, latency from peak = 15±5ms, ±SD). During bouts of singing, Uva activity is persistently elevated and is strongly modulated in a manner locked to song (Fig 4A). One of the most prominent features of Uva activity during singing is the robust activation prior to each introductory note (Fig 4B, latency from baseline = 39±8ms, peak latency = 21±9ms, ±SD). Uva activity also shows a pattern of modulation locked to song syllables and these modulations are consistent across repetitions of the song motif (Fig 5A); the smoothed multiunit signals are highly correlated across different song renditions (0.64±0.06 with 5ms smoothing) and coherent across different song renditions at frequencies below 12Hz (C_avg_ = 0.68±0.09 between 1-12Hz, ±SD) (Fig 5B and 5C).

To examine the spatial homogeneity of multiunit activity within Uva, recordings were made sequentially at different depths along the same penetration (Fig 5D). We find that activity at different recording sites throughout Uva is also highly correlated (0.62±0.03 with 5ms smoothing) and coherent (C_avg_ = 0.65±0.13 between 1-12Hz, ±SD) (Fig 5E and 5F). In summary, we find no evidence that Uva activity varies across recording sites.

**Fig 6:**
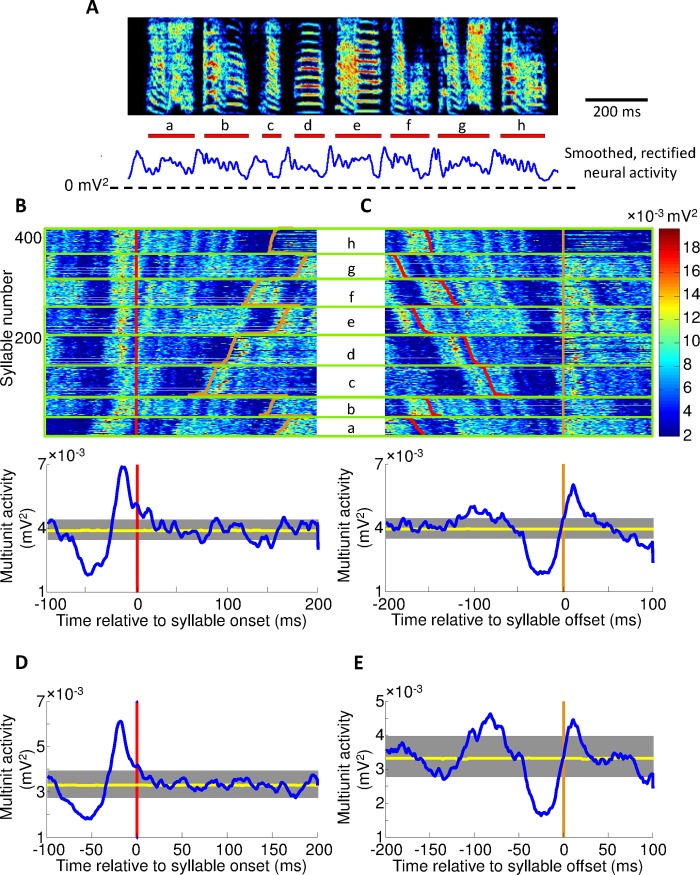
Uva activity peaks prior to syllable onset and dips prior to syllable offset. (**A**) Above is a spectrogram of a single motif. Red bars represent the syllable lengths, with syllable labels below. Also showed is the trial-averaged smoothed, rectified neural activity in Uva. The song shown here is the same as that shown in Fig 5A **(B)** Uva activity peaks prior to syllable onset. Raster(top) represents the power of neural activity during each syllable rendition. Red line marks syllable onset and white line marks syllable offset. Syllables are grouped based on identity, arranged from longest to shortest syllable in descending order and then aligned to syllable onset. Individual syllables have been identified and labeled. Below is a syllable onset aligned multiunit trace averaged across all syllables. Also shown is the 95% confidence interval of baseline activity during vocalization determined from random shuffling of multiunit activity (yellow trace). **(C)** Uva activity dips prior to syllable offset. Heat raster (top) shows all syllables aligned to syllable offset. Average trace (below) shows a dip prior to syllable offset. Black line represents syllable offset. **(D)** Syllable onset aligned multiunit trace averaged across all birds. **(E)** Syllable offset aligned multiunit trace averaged across all birds.

**Fig 7:**
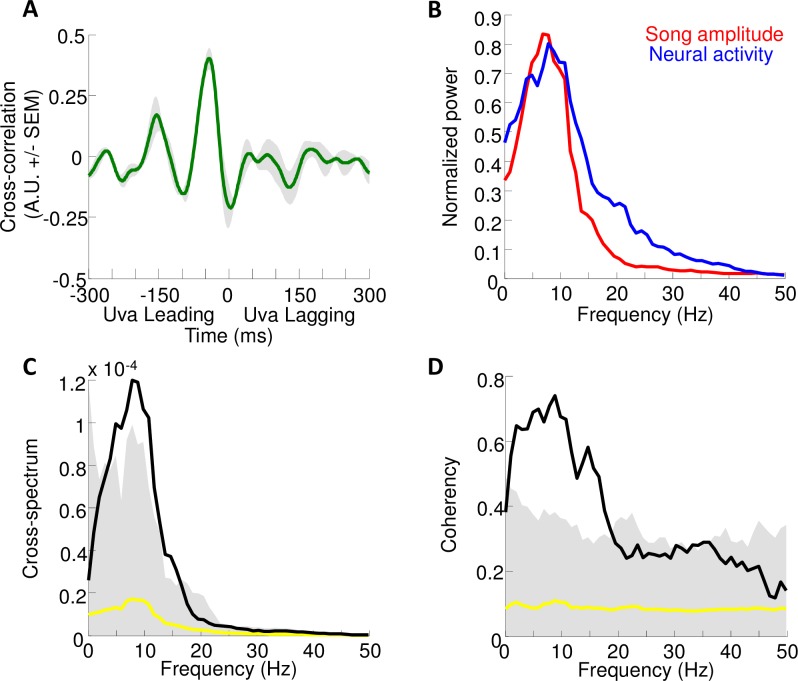
Quantification of rhythmic activity in Uva. **(A)** Cross‐correlation function between Uva activity and sound amplitude averaged across 5 birds (green: mean cross-correlation, shaded region: SEM; peak correlation = 0.40±0.04, mean lag at peak correlation = 42±6ms). **(B)** normalized power spectra of the song amplitudes (red) and Uva multiunit activity (blue), averaged across n = 5 birds. A broad peak in the power spectrum is seen in both the song amplitude and neural data, centered ~10Hz. **(C)** Cross-spectrum between Uva activity and sound amplitude averaged across n = 5 birds (black: mean, yellow: null cross-spectrum, shaded region: 95% percentile corrected for multiple testing) **(D)** Coherency between Uva activity and sound amplitude averaged across n = 5 birds (black: mean, yellow: null cross-spectrum, shaded region: 95% percentile corrected for multiple testing). Note a large, significant peak is observed in both the cross-spectrum and the coherence at ~10Hz (F_peak_ = 8.8Hz, C_peak_ = 0.75, p<0.01, phase = -0.77π).

Multiunit activity in Uva is strongly related to syllable patterning (Fig 6A), exhibiting a significant increase in activity prior to syllable onsets (p<0.01 paired t-test), and a significant decrease prior to syllable offsets (Fig 6B; significant for each bird recorded; n = 5). Uva activity peaks 20±2ms (±SEM) prior to syllable onsets, and rises significantly above the average level of activity during singing 32±5ms before syllable onsets (Fig 6C; quantities averaged across all birds recorded; n = 5). The decrease in Uva activity at syllable offsets reaches a minimum 18±1ms (±SEM) prior to the offset (Fig 6D) and drops significantly below average Uva activity during singing 48±8ms prior to the offset (see Methods) (data from additional birds shown in S2 and S3 Figs). Uva activity is also strongly correlated with song amplitude (magnitude of peak correlation: 0.40±0.04), with a latency of 42±6ms (Fig 7A, ± S.D).

We wondered whether variations in Uva activity may correlate with variations in song features within syllables [49]. We analyzed the correlation between Uva multiunit activity and six commonly used spectral features (pitch goodness, Weiner entropy, amplitude, amplitude modulation, frequency modulation, and gravity center). Song spectra features were calculated in windows from 10ms after syllable onsets to 10ms prior to syllable offsets. We took into account the latency of Uva activity, as calculated from the correlation with song amplitude (42ms). None of these six spectral features is significantly correlated with Uva activity in individual birds. When combined across all birds, only amplitude (r = 0.18) and gravity center (r = 0.17) exhibit significant correlation after Bonferroni correction for multiple comparisons (6 comparisons; p<0.008).

While most adult zebra finch song is not highly rhythmic, it has been reported that these songs can contain an underlying rhythm in the 10 Hz range [45]. Indeed, we found that the song amplitude profile of our adult birds exhibits a broad spectral peak near this frequency (Fig 7B; peak of average song rhythm spectrum at 8.0Hz). Notably, Uva multiunit activity also exhibits peaks at nearly the same frequency (8.8Hz). Further analysis reveals a large peak in the cross spectral density between multiunit activity and song amplitude, as well as a significant coherency at this frequency (F_peak_ = 8.8Hz, C_peak_ = 0.75, p<0.01, phase = -0.77π) (Fig 7C and 7D). The pronounced coherence at approximately 10Hz suggests that rhythmic modulations in song amplitude are correlated with modulations in Uva activity.

**Fig 8:**
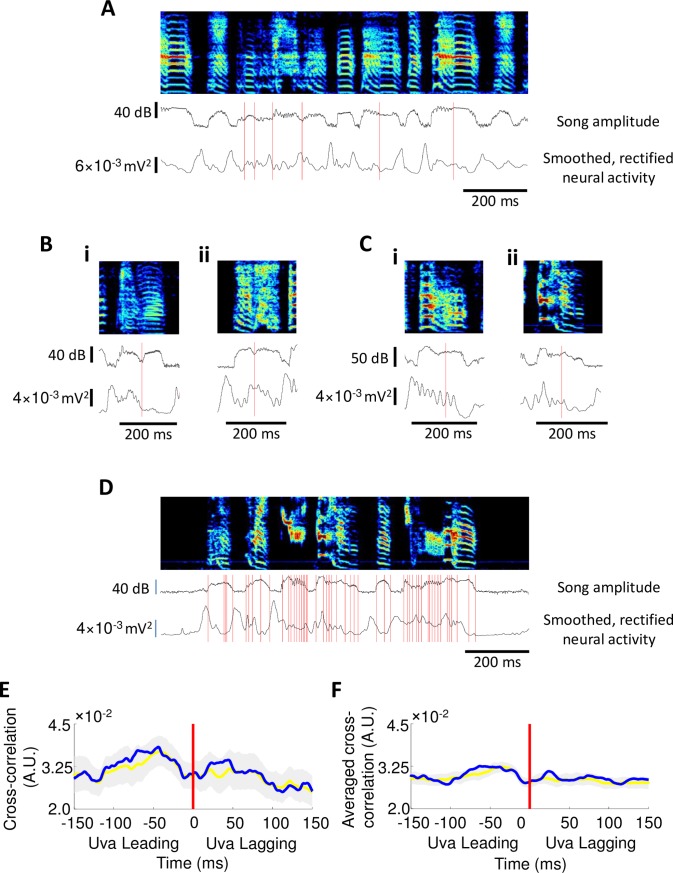
Uva activity exhibits no significant correlations with acoustic transitions or gesture trajectory extrema (GTEs). (**A**) Many syllables in a song may exhibit one or more acoustic transitions. Red lines mark acoustic transitions within long syllables. Trial-averaged song amplitude and smoothed, rectified neural activity also shown. (**B**) Examples of syllables where peaks in Uva activity are associated with acoustic transitions. Trial-averaged song amplitude and smoothed, rectified neural activity also shown. (**C**) Examples of syllables where acoustic transitions are not associated with peaks in Uva activity. Trial-averaged song amplitude and smoothed, rectified neural activity also shown. (**D**) GTE were identified using automated algorithm. Red lines mark GTEs that were identified by the automated algorithm from *Boari et al*. Also shown is a trial-averaged song amplitude and smoothed, rectified neural activity. (**E**) Uva activity does not exhibit any significant correlation with GTE times in individual birds and (**F**) across all birds when compared to a surrogate datasets in which GTE times were randomly shuffled within song syllables (gray shading: 99% confidence interval of surrogate dataset distribution; yellow: surrogate dataset distribution; blue: cross-correlation between Uva activity and GTE times).

While Uva activity appears to be strongly associated with syllable onsets and offsets, significant modulations are also observed within song syllables, particularly long, multi-part syllables. In some complex syllables, such peaks in Uva activity appear to be associated with some acoustic transitions identified manually (Fig 8A and 8B), but the relation between Uva activity and such transitions is not reliable (Fig 8C). To more quantitatively analyze this relation, we examined how neural activity varies relative to extrema of control parameters inferred from song (GTEs) [31]. Using a previously published automated method to identify GTEs [50] (Fig 8D), we identified GTE times in the song and calculated the cross correlation between GTEs and Uva multiunit activity. No significant peaks are observed in this correlation within individual birds or averaged across birds, compared to surrogate datasets in which GTE times are randomly shuffled within song syllables (Fig 8E and 8F).

**Fig 9:**
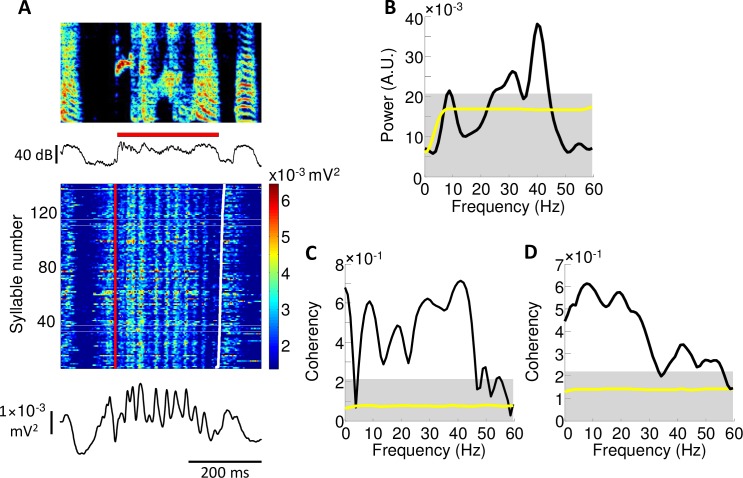
Rhythmicity during long syllables. (**A**) In many long syllables (>150ms in length), we observed rapid oscillations in Uva activity. (**B**) In these long syllables, we consistently observed a peak in the power spectrum of Uva activity at frequencies in the gamma range. (**C**) Uva activity during these long syllables is significantly coherent with song amplitude across a large frequency range (1-55Hz) and (**D**) across all birds when compared to null distribution (yellow), calculated from randomly shuffled neural data (see Methods) (gray shading indicates 95% confidence interval for maxima anywhere in this window).

While Uva activity across birds reliably exhibits a 10Hz rhythm, we also observe in some syllables a modulation in Uva activity at frequencies in the gamma range (Fig 9A). Such rapid modulations are pronounced in some syllables, but are entirely absent in others. To further quantify this phenomenon, we performed spectral analysis on individual syllables (analysis done for 13 syllables longer than 150ms, in order to provide adequate sample duration; this was 43% of all song syllables). Of these 13 syllables, 8 syllables show a significant peak in the spectrum of the neural activity greater than 10Hz. These peaks occur at frequencies ranging from 17 to 51Hz. Notably, in individual syllables, Uva activity and sound amplitude are found to be significantly coherent across a broad range of frequencies (1-55Hz) (Fig 9C and 9D), indicating that, even at these higher frequencies, Uva activity appears to be significantly correlated to variations in song amplitude (S4 Fig).

Finally, we find little evidence for activation in Uva related to the onsets or offsets of longer timescale song structures, such as song motifs or bouts, as has been previously reported [26]. We did find, however, that Uva activity was transiently suppressed following bout offsets for a period of approximately 200ms (S5 Fig).

## Discussion

Using a combination of behavioral and electrophysiological studies, we re-examined the role of Uva in the production of stereotyped adult song. Our lesion experiments largely support the conclusion of earlier lesion studies indicating a prominent role for Uva in adult song production [26,27]. We have additionally examined this question for singing in the directed and undirected context. Similar to the earlier studies, we find that in the directed social context, birds with complete bilateral Uva lesions are unable to sing. However, we also find that, in the undirected context, birds with complete bilateral lesions of Uva are still able to sing, but the song suffers a loss of stereotyped acoustic and temporal structure; in particular the undirected song had a nearly exponential distribution of syllable durations, characteristic of early vocal babbling [41,44].

This pattern of effects of Uva lesions on directed and undirected song is similar to that previously reported for birds with bilateral HVC lesions [3,41,43,44]. Like bilateral Uva lesions, bilateral lesions of HVC profoundly alter adult singing behavior, but have differential effects on directed and undirected song. First, HVC lesions completely abolish directed song. Specifically, when presented with a female, HVC-lesioned birds approach the female and appear to attempt singing, but no vocalizations are produced [3,43]. In contrast, birds with HVC lesions can still produce song in social isolation (undirected song), but their vocalizations lack the stereotyped structure of normal adult song. In fact, the undirected song produced by HVC-lesioned birds is highly variable and resembles subsong, the most juvenile ‘babbling’ form of singing, and is likely generated by inputs to the motor pathway from the cortical nucleus LMAN [41,43,51]. Our observations suggest that lesions of Uva are functionally similar to HVC lesions, consistent with the idea that Uva plays a role in activating HVC during singing.

A recent family of hypotheses attempts to relate neural activity within the song system to behavioral subunits of song defined by the onset, offsets, and extrema of vocal control parameters. These discrete behavioral events are collectively called GTEs (Gesture Trajectory Extrema) [31,50]. The GTE model suggests that GTEs are sparsely encoded by an unidentified brainstem pattern generator, called the initiating area (IA), that drives brainstem vocal-respiratory motor systems [32]. In addition to activating vocal-respiratory systems, the IA is hypothesized to activate HVC at the times of GTEs via Uva. Thus a prediction of this model is that Uva will display sparse bursts at the times of GTEs. Our recordings reveal a peak in Uva activity associated with syllable onsets, consistent with this hypothesis. However, the GTE model also also predicts a peak in Uva activity at syllable offsets and at GTEs within syllables; instead our recordings reveal a pronounced dip at syllable offsets and no consistent relation between Uva activity and intra-syllable GTEs. In summary, our findings do not support the GTE model as currently elaborated [31,32]. Recent recordings in HVC have also failed to support the GTE hypothesis [52,53].

It has been proposed that the continuous activity in HVC is mediated by a synaptically connected chain of neurons [12,37,38]. According to this hypothesis, known as the chain model, activity could propagate [54–56] through the HVC network—like a chain of falling dominoes—forming the basic clock that underlies song timing. The chain model is supported by several lines of evidence. First, mild cooling of HVC, but not RA, slows the song, suggesting the dynamics controlling song timing exist primarily within HVC [34]. Second, intracellular recordings during singing found that the subthreshold membrane potentials of HVC neurons are characterized by a large, rapid depolarization 5–10 ms before burst onset, consistent with activation by earlier nodes in a chain [12], and inconsistent with a role for slow intracellular dynamics in sequence generation. Finally, recent studies of the distribution of bursts in HVC suggest that these events occur with a nearly uniform distribution throughout song and provide complete coverage in time [52,53].

In the simplest form of the chain model, the entire motif could be generated by one long chain in HVC. However, several lines of evidence suggest that the motif is not generated by a single continuous chain but rather by multiple discrete chains, potentially associated with syllables [12,39,40]. For example, bilateral, multiunit recordings in HVC reveal brief periods of interhemispheric synchronization related to syllable onsets, suggesting a modular organization of HVC at the level of syllables [28]. Further support for this view comes from several different observations: 1) a detailed analysis of song timing showing that the durations of silent gaps between syllables are more variable than the durations of syllables [57], 2) flashes of light cause the interruption of syllables with high probability at the ends of syllables [58], and 3) local cooling of HVC has a different effect on respiratory patterns in syllables versus gaps [40]. Overall, these results suggest that the links between song syllables are mediated by a different mechanism than the structure within syllables.

It has been hypothesized that the link between HVC sequences corresponding to different syllables may be mediated by the feedback loop through the thalamic nucleus Uva [29,34,39] In this feedback loop model, the end of one chain of neurons in HVC activates Uva, which in turn initiates the next chain in HVC prior to the next syllable onset. It has also been suggested that activity in this midbrain-thalamic feedback loop may mediate the bilateral global synchronization of the two HVCs during singing [28,59,60]. At the simplest level, one might expect under this hypothesis that the peak in Uva activity prior to syllable onsets should precede activity in HVC. Despite the continuous coverage of HVC bursts throughout song, a recent study has described a weak syllable-related modulation in projection neuron burst density and interneuron firing rates. Indeed, we found that the peak in Uva activity occurs slightly before the peak in HVC burst density [53] (20±2ms versus 18±2ms, respectively). While this latency difference may be surprisingly small, we should note that the antidromic latency from Uva to HVC is extremely short (~1.5ms). Furthermore, if HVC responds most strongly to the rising phase of the syllable-onset activity in Uva, this could cause the peak in HVC activity to occur at nearly the same time as that in Uva. Thus, our findings are consistent with the hypothesis that Uva activates chains in HVC prior to syllable onsets.

Similar to what we found in Uva, a rhythmic modulation of neural activity coherent with a ~10Hz rhythm in song structure was previously seen in adult and juvenile HVC[47,53]. This observation has led to the hypothesis that these oscillations play a role in song development. In zebra finches, the adult song motif emerges during learning from an earlier stage of song development in which primitive ‘prototype syllables’ are rhythmically repeated at 10 Hz [43,45–48]. During this early stage, HVC projection neurons also generate bursts with significant 10Hz rhythmicity locked to song syllables. It is possible that the 10Hz rhythmic activity we observe in the adult song circuit is a vestigial remnant of the early stages of song development. Alternatively, it is possible that the avian song system is functionally organized around a 10Hz rhythm, with multiple chains in HVC that span ~100ms period. According to this view, the fundamental ‘unit’ of song would not be the syllable but, rather, one cycle of the 10Hz rhythm. This view might also account for observations indicating that many long complex syllables may be composed of smaller underlying units [28,58].

While our findings are broadly consistent with the idea that Uva serves to bilaterally activate sequences in HVC prior to syllable onsets, its role in other aspects of song production remain unclear. For example, individual HVC-projecting Uva neurons could, in principle, be highly selective for individual syllable types, perhaps controlling song syntax by selectively activating particular syllable chains in HVC. Alternatively, these neurons could be active, in a non-selective way, before every song syllable, and serve simply to synchronize the two hemispheres by simultaneously initiating HVC sequences. Finally, it remains a possibility that Uva could simply supply the excitatory tone necessary for HVC to function [61], without having a role either in selecting or initiating HVC sequences. Previous studies have reported that partial unilateral or bilateral Uva lesions disrupt the stereotyped order of syllables within motifs [26], suggesting Uva may control song syntax. On the other hand, our recordings reveal a remarkable degree of homogeneity in firing patterns at different sites within Uva, possibly hinting at a high degree of homogeneity among Uva neurons, and thus favoring a model in which Uva does not play a role in selecting syllable types. Single unit recordings of identified HVC-projecting Uva neurons will ultimately be required in order to differentiate these different models.

Recently, an alternative model has been proposed which argues that song timing is generated by a distributed and recurrent network that spans the avian forebrain and brainstem, of which HVC is a component [25,62]. Thus, while chain model proposes that sequential activity in HVC largely results from propagation of burst activity through synaptic connected chains of neurons within HVC—with the possible exception of connections between syllables through the brainstem-thalamic feedback loop—this new model proposes that all sequential activity in HVC results from rapid cycling of activity through the brainstep-thalamic feedback loop. In this model, each burst in HVC is driven by a temporally distinct input from Uva. Note that the rapid cycling model still incorporates a chain, but this chain propagates through Uva rather than within HVC. The rapid cycling model makes several predictions regarding Uva activity during song: first, that activity in Uva should be consistently active throughout song and should exhibit no gaps, and second, that Uva activity encodes each moment in the song uniquely in order to drive the next unique sparse state in HVC. Neither of these predictions is supported by our observations; the pause in Uva activity observed prior to the end of each syllable offset would not enable propagation of burst activity from the end of one syllable to the onset of the next. Second, the highly correlated patterns of activity at different recording sites within Uva are not consistent with Uva maintaining a temporally unique representation for each time in the song. Both of these predictions of the rapid-cycling model would also be better assessed using single-unit, rather than multi-unit, recordings in Uva.

The high degree of homogeneity in firing patterns at different sites within Uva also raises questions regarding the function of different Uva neuron types during song. Uva has known projections to three telencephalic song nuclei: NIf, HVC, and an auditory cortical area Avalanche (Av). These projections arise from at least two different, spatially intermingled neuron types [22]. Unfortunately, our multiunit recordings do not allow us to distinguish between these different projection pathways, and single unit recordings of antidromically identified Uva neurons will ultimately be required to determine what the role of these neuronal populations in adult song production.

While all neocortical areas receive thalamic inputs, the functional role of the thalamus in driving the cerebral cortex, particularly areas beyond the primary sensory cortices, remains poorly understood. In the sensory system, the classical view of the thalamus, in particular first order thalamic nuclei, is that of a relay station transmittin peripheral sensory information from the external world to the cortex [63,64]. The same may also be true about movement information. The execution of complex motor tasks requires both the generation of movements as well as monitoring of those generated movements. Information about movements can originate from both sensory receptors (peripheral reafference) [65,66], including those in muscles, as well as from internal representations of those movements, known as either collateral discharge or efference copy [67,68]. It is likely that much of the information the brain receives regarding self-generated movements is relayed to the cortex via the thalamus [69].

Information relayed by the midbrain-thalamic feedback loop may be necessary in the initiation and sequencing of behavioral units into a single, cohesive behavior. Like the midbrain-thalamic feedback loop in songbirds, a similar ascending connection between a subcortical motor center and a premotor cortical center exists in primates. The circuit, consisting of the superior colliculus (SC) which projects to the frontal eye field (FEF) via the mediodorsal thalamus (MD), is involved in the generation of saccadic eye movements. A combination of behavioral [70] and electrophysiological [71] studies suggest that this midbrain-thalamic feedback pathway in primates relays a corollary discharge of midbrain motor output that is used for coordinating sequential saccades and possibly for stabilizing vision across saccades. The avian midbrain-thalamic feedback loop may act in an analogous manner. It has been hypothesized that the activity in Uva carries an efference copy, or corollary discharge, of respiratory output from the brainstem during singing [24]. For example, the persistent activity in Uva during syllables may reflect persistent respiratory drive during syllables, while the dips in Uva activity prior to syllable offsets may reflect the drop in expiratory drive prior to the initiation of an inspiratory pulse. We hypothesize that such a respiratory efference copy signal serves to sequentially activate syllables during singing. Overall, these findings in the primate and avian brain are consistent with a general model for how the brain coordinates the sequential structure of complex behaviors, namely that thalamic feedback serves as a means for the cortex to track what movements have been performed so that the next component of the behavior can be initiated appropriately [69].

In conclusion, our results are consistent with earlier work showing that Uva is necessary in the production of stereotyped, adult song, and shed further light on the role Uva plays in song production. Uva occupies a strategic position that allows it to coordinate activity in premotor brain areas across hemispheres and, also to activate the next behavior in a complex sequence. Although future electrophysiology and other experimental procedures are needed to provide greater insight into the precise role of Uva in the production of adult song, our results suggest that the thalamic nucleus is critical in patterning adult vocalizations.

## Materials and methods

Subjects were adult male zebra finches, >90 days post hatch (dph). Birds were obtained from either the Massachusetts Institute of Technology breeding facility or a commercial breeder. Animal care and experiments were performed in accordance with the National Institute of Health guidelines and approved by the Massachusetts Institute of Technology Institutional Committee on Animal Care.

### Sound recordings

Several days prior to surgery, birds were placed in custom-made sound isolation chambers. Vocalizations were recorded with custom-written Matlab software or with Sound Analysis Pro, which were configured to record the soft vocalizations of subsong.

### Antidromic identification of Uva

Uva was localized by antidromic stimulation from HVC. Before surgery, anesthesia was induced with 1–3% isofluorane in oxygen. After mapping out HVC as described below, a bipolar stimulating electrode was implanted in HVC for antidromic identification of Uva. Single monopolar pulses of 0.2ms duration was produced using an isolated stimulation unit (AMPI, Inc) controlled by a Master 8 (AMPI, Inc), with intensities varying from 50–200μA. Uva neurons were found using ongoing 1 Hz stimulation in HVC to elicit spike responses.

### Localization of HVC

We localized HVC by antidromic stimulation of Area X. A bipolar stimulating electrode was implanted into Area X using stereotaxic coordinates (Head Angle: 0°, AP: 5.40, ML:1.50, DV:-2.80). HVC neurons were identified by an ongoing 1Hz monopolar pulse of 0.2ms duration, with intensities varying from 50–200μA. After localizing HVC, a retrograde tracer (dextran) was injected into HVC in order to label HVC-projecting Uva neurons.

### Lesions

The location of Uva was identified and mapped by antidromic stimulation in HVC. After Uva was located, electrolytic lesions were made using a 1MΩ Pt-Ir electrode (MicroProbes, PI20031.0A3). To ensure a complete lesion, approximately -2μA of current was passed for 60 s, usually at two locations 150μm apart along the anterior-posterior axis. Prior to implantation of the stimulating electrode in HVC, a retrograde neuronal tracer (dextran) was injected into HVC bilaterally to permit later assessment of the extent of Uva lesion. After surgery, the birds were allowed to recover from surgery and then placed back into the sound isolation chambers. Birds typically began to sing again 1–3 days post-surgery. Both directed and undirected song was recorded for up to a week beginning from the first day of singing post-surgery.

### Chronic neural recordings in Uva

Experiments were carried out using a motorized microdrive as previously described [72,73]. The microdrive weighed ~1.5g and contained a single microelectrode (MicroProbes, PI20035.0A3 5MΩ ~5-10um tip length). Microdrives were implanted in the right hemisphere of all the birds used in this study. As the bird sang, the electrode was advanced slowly throughout the dorsal-ventral extent of Uva. A small lateral positioner allowed us to displace the electrode by several tens of micrometers in order to make a fresh penetration through Uva. The HVC-projecting neurons of Uva were identified by antidromic stimulation via HVC. We were able to record single units in Uva under anaesthesia and in awake, non-singing birds. However, we found that during singing, Uva neurons spiked at very high rates making single unit isolation impossible. On the final day of recording, the recording electrode was retracted ~200μm above Uva and an electrolytic lesion was made through the recording electrode (-15μA for 15sec) allowing histological confirmation of the placement of the electrode tip.

In order to eliminate movement artifact, recorded signals were passed through a headstage source-follower amplifier consisting of a field-effect transistor (FET) (2SK3796-3-TL-E, ON Semiconductor). This decreases movement artifact generated by flexion of the cable. Electrode signals were band-pass filtered in hardware between 300Hz and 15kHz (Texas Instruments TL084ACN and Frequency Devices D68L8E, respectively) and amplified by a factor of 1000 before digitization. Audio and electrophysiology were digitally sampled at 40kHz [72,73]. Multiunit activity was quantified from the recorded signal by rectifying and smoothing with a 2 ms (SD) Gaussian function.

### Histology

Following the last day of recordings, birds were euthanized with FatalPlus and perfused transcardially with 0.2 M phosphate-buffered solution followed by 4% paraformaldehyde in phosphate-buffered solution. Brains were post-fixed overnight and cut into 100 μm thick sagittal sections on a vibratome. Sections were stained for the neuronal marker NeuN (Millipore, A60) and mounted. Uva lesions were confirmed by the absence of retrogradely-labeled HVC-projecting cells in the thalamus. Proper injection of retrograde tracer into HVC was confirmed by the presence of retrogradely labeled cells in nucleus interface (NIf).

### Sound analysis

All data analyses were performed with custom MATLAB software. Syllables and gaps were segmented based on the analysis described by Aronov et al., 2011. The audio signal was preprocessed with a 1–4 kHz bandpass filter. The sound amplitude was determined by squaring the audio signal and smoothing it with a 2.5ms (SD) Gaussian function. The relative sound level was converted to decibels by taking the logarithm (base 10) of the processed audio signal and multiplying it by 10. Sound amplitude produced during singing is bimodally distributed, corresponding to vocalized syllables and silent gaps. The mean and SDs of these two mode were estimated by fitting two Gaussian curves to the sound level distribution using expectation maximization.

For syllable segmentation in each recording, we calculated a sound threshold as the Fisher discriminant of two Gaussian modes (corresponding to noise and sound) fit to the values of log-amplitude. We detected crossings of this threshold and defined sound onsets and offsets as the closest points to these crossings where amplitude deviated from noise by 2 standard deviations. Sounds separated by <7 ms of silence were merged into a single syllable, and segments of sound <7 ms long were eliminated. Bouts were defined as a sequence of syllables with gaps no longer than 300ms. Syllable renditions with noise or female calls were removed from the analysis. All syllable onsets and offsets were manually verified for accuracy.

To quantify the extent to which Uva-lesioned song resembles subsong, we carried out an analysis of the distribution of syllable durations [41]. Syllables and gaps were initially analyzed by fitting an exponential function to their duration distribution using maximum-likelihood estimation (MLE). This analysis was performed on song data collected during one day of singing, and consisted of 1000–10,000 syllables. The goodness-of-fit (Γ) of the exponential was estimated using the Lilliefors statistics [42]. Distributions that are similar to subsong and are well fit by the exponentials typically have a goodness-of-fit metric <2. Distributions similar to early plastic song and are beginning to exhibit a protosyllable peak, typically have values >2.

### Song rhythmicity

Song rhythmicity was determined according to the analysis described by Saar et al., 2008. To compute song rhythm, we first extracted the sound amplitude during song bouts. Bouts were defined as continuous stream of syllables with gaps no more than 350ms. The sound amplitude within each bout was rectified, smoothed, and converted to a log scale. The sample was then mean-subtracted and detrended. Finally, the spectrum of the song amplitude was computed using the FFT function in Matlab (1 tapers, 2 time half-bandwith product). The frequency spectrum was then normalized by bout length and squared to obtain the power spectrum. Song rhythmicity *R* was quantified as the maximum of the ratio between the normalized power spectrum and the null power spectrum at frequencies greater than 3 Hz. In this case, the null power spectrum was generated from an exponential distribution of syllable durations and a unimodal distribution of gap durations, which is seen in subsong [44]. Only peaks above 3 Hz were considered because these correspond to the typical frequency at which syllables occur during singing [45].

### Maturity index

To quantify the level of stereotypy, we used the analysis described by Aronov et al., 2008 based on a spectral correlation of different bouts produced by the same bird. Adult song is highly stereotyped and thus exhibits a high degree of spectral correlation across renditions. In contrast, young birds exhibit much less stereotyped song and exhibit a lower degree of correlation across song bouts. Approximately 100 bouts were randomly selected from the data. We only considered bouts that were, at least, 700 ms long and at most 2 s long. These bouts thus included at least two song motifs. Spectrograms were calculated using the multi-taper method (2 tapers, 10 ms window, 1 ms step size, bandwidth parameter of 1.5); [49]. For all possible distinct pairs of bouts in this data set, a correlation matrix was calculated by computing the correlation of power spectra between 860 Hz and 8.6 kHz for each pair of 1 ms time slices of the spectrogram. We then measured the maximum value of the lag correlation function. The resulting value was averaged over the ~10,000 comparisons.

### Time warping for song alignment

The duration of song motifs of zebra finch song can vary from bout to bout by up to 9ms. [74]. This jitter can introduce considerable noise to the structure of multiunit activity in Uva if each bout is aligned only to song motif onset. To display the neural activity in Uva aligned to a single song motif, we time warped the multiunit activity using syllable onsets and offsets in the motif as alignment points [75]. Multiunit activity between each alignment point was then either stretched or compressed to match the corresponding interval in a representative template motif. To select the representative template motif, we determined the median motif length and chose the bout whose length is closest to that value. This piecewise linear time warping was based on the song structure and was independent of the multiunit activity.

### Gesture Trajectory Extrema (GTE) analysis

GTE times were extracted from the songs using a previously published automated method [50]. The approach is to use a dynamical model of the vocal organ (the syrinx) to infer the trajectory of two control parameters—air sac pressure and labial tension [32,76]. Continuous segments of control parameters are called ‘gestures’, and local maxima in either of the two control parameters within a gesture are called extrema. These, together with the beginning and end of the gesture, are identified as gesture trajectory extrema, or GTEs.

To calculate the null distribution for the cross-correlation between Uva activity and GTE times, the total number of GTEs was redistributed probabilistically across syllables based on syllable length. After redistributing GTEs among the different syllables, the GTEs were redistributed within syllables randomly. GTEs occurring at syllable onset and offset were kept at their calculated times based on the previously described algorithm.

### Rhythmicity

The spectral analysis of the song amplitude and multiunit activity was carried using code from the Chronux package [77]. Quantities calculated include power spectral density, cross power spectral density, and coherency (1 tapers, 2 time half-bandwith product). Digitized neural activity and sound amplitude waveforms were rectified, and smoothed with a 2ms (SD) Gaussian function. The null distributions for coherency and cross-spectrum were determined by randomly shifting multiunit activity relative to song amplitude for all renditions, averaged across 1000 trials.

For the analysis of long syllables, we first selected syllables of lengths greater than 150ms. We then performed spectral analysis on the neural activity and song amplitude from syllable onset to 50ms prior to syllable offset. This was done in order to exclude the peak in Uva activity prior to syllable onset and the dip in Uva activity prior to syllable offset. The power spectrums were normalized by the sum of the power spectrum. To calculate the null distribution of the neural power spectrum, Uva activity was randomly scrambled then smoothed with a 2ms (SD) Gaussian function. This null data set was then processed using the spectral analysis techniques described above.

## Supporting information

S1 Video Changes in adult bird singing behavior following Uva lesions. In the context of directed singing, lesioned birds demonstrated typical courtship behaviors, including approach and bill wiping. However, lesioned birds failed to sing and only produced sporadic short sounds, acoustically similar to introductory notes but without their characteristic rhythmicity.
(RAR)

S1 Fig Song stereotypy is unaffected by lesions in brain regions surrounding Uva. **(A)** (left) Retrograde tracers from HVC (dextran-conjugated Alexa Fluor 480) were used to distinguish Uva-HVC projectors from surrounding thalamic neurons (Neu-N, red) in an intact bird (yellow scale bar = 200μm). (middle, right) Absence of Neu-N stain reveals bilateral elimination of brain regions surrounding Uva. Dotted-line marks the border of Uva. (*Inset)*retrograde labeling from HVC in intact NIf demonstrate successful retrograde tracing (scale bar = 200μm) **(B)** (top) Prelesion song spectrogram of an adult bird (>90dph). Bottom trace is the song amplitude and the black segments indicate individual syllables. (Bottom) song spectrogram of the same bird taken from the first day of singing after bilateral control lesions. Note the song stereotypy in the duration of syllables and gaps between syllables, as well as the acoustic features of the song remains largely intact. **(C)** and **(D)** Distribution of syllable and gap durations, respectively, before (black trace) and after(red trace) bilateral control lesion. The null distribution for syllables and gaps is represented by an exponential or unimodal distribution, respectively (dashed blue trace). **(E)** normalized power spectra of the song amplitudes before and after lesion. Null power spectrum distribution (dotted blue) was generated from an exponential distribution of syllable durations and a unimodal distribution of gap durations.
(TIF)

S2 Fig Uva activity peaks prior to syllable onset and dips prior to syllable offset. Above is a spectrogram of a single motif. Red bars represent the syllable lengths, with syllable labels below. **(A)** (**i**) Uva activity peaks prior to syllable onset. Raster(top) represents the power of neural activity during each syllable rendition. Red line marks syllable onset and orange line marks syllable offset. Syllables are grouped based on identity, arranged from longest to shortest syllable in descending order and then aligned to syllable onset. Individual syllables have been identified and labeled. Below is a syllable onset aligned multiunit trace averaged across all syllables. Also shown is the baseline activity during vocalization determined from random shuffling of multiunit activity (yellow; shading indicates 95% confidence interval for maxima and minima anywhere in this window). **(ii)** Uva activity dips prior to syllable offset. Heat raster (top) shows all syllables aligned to syllable offset. Average trace (below) shows a dip prior to syllable offset. (**B**) Data from an additional bird.
(TIF)

S3 Fig Uva activity peaks prior to syllable onset and dips prior to syllable offset. Above is a spectrogram of a single motif. Red bars represent the syllable lengths, with syllable labels below. **(A)** (**i**) Uva activity peaks prior to syllable onset. Raster(top) represents the power of neural activity during each syllable rendition. Red line marks syllable onset and orange line marks syllable offset. Syllables are grouped based on identity, arranged from longest to shortest syllable in descending order and then aligned to syllable onset. Individual syllables have been identified and labeled. Below is a syllable onset aligned multiunit trace averaged across all syllables. Also shown is the baseline activity during vocalization determined from random shuffling of multiunit activity (yellow; shading indicates 95% confidence interval for maxima and minima anywhere in this window). **(ii)** Uva activity dips prior to syllable offset. Heat raster (top) shows all syllables aligned to syllable offset. Average trace (below) shows a dip prior to syllable offset. (**B**) Data from an additional bird.
(TIF)

S4 Fig Rhythmicity during long syllables. (**A**) In many long syllables (>150ms in length), we observed rapid oscillations in Uva activity. (**B**) In these long syllables, we consistently observed a peak in the power spectrum of Uva activity at frequencies in the gamma range. (**C**) Uva activity during these long syllables is significantly coherent with song amplitude across a large frequency range (1-55Hz) and (**D**) across all birds when compared to null distribution (yellow), calculated from randomly shuffled neural data (see Methods) (gray shading indicates 95% confidence interval for maxima anywhere in this window).
(TIF)

S5 Fig Uva activity is suppressed following bout offset for approximately 200ms. (**A**) A bout offset (blue) aligned multiunit trace averaged across all bouts (black). Also shown is the baseline activity during non-singing determined from random shuffling of multiunit activity (yellow; shading indicates 95% confidence interval for maxima and minima anywhere in this window).
(TIF)

## References

[1] Lashley KS. The problem of serial order in behavior. Bobbs-Merrill; 1951 112–136 p.

[2] Zann RA. The zebra finch: A synthesis of field and laboratory studies. Oxford University Press; 1996.

[3] Nottebohm F, Stokes TM, Leonard CM. Central control of song in the canary, Serinus canarius. J Comp Neurol [Internet]. 1976/02/15 ed. 1976;165(4):457–86. Available from: http://www.ncbi.nlm.nih.gov/entrez/query.fcgi?cmd=Retrieve&db=PubMed&dopt=Citation&list_uids=1262540 10.1002/cne.901650405 1262540

[4] Bottjer SW, Halsema KA, Brown SA, Miesner EA. Axonal connections of a forebrain nucleus involved with vocal learning in zebra finches. J Comp Neurol [Internet]. 1989/01/08 ed. 1989;279(2):312–26. Available from: http://www.ncbi.nlm.nih.gov/entrez/query.fcgi?cmd=Retrieve&db=PubMed&dopt=Citation&list_uids=2464011 10.1002/cne.902790211 2464011

[5] Vu ET, Mazurek ME, Kuo YC. Identification of a forebrain motor programming network for the learned song of zebra finches. J Neurosci [Internet]. 1994/11/01 ed. 1994;14(11 Pt 2):6924–34. Available from: http://www.ncbi.nlm.nih.gov/entrez/query.fcgi?cmd=Retrieve&db=PubMed&dopt=Citation&list_uids=7965088 7965088 10.1523/JNEUROSCI.14-11-06924.1994 PMC6577280

[6] Karten HJ. Homology and Evolutionary Origins of the “Neocortex.” Brain Behav Evol [Internet]. 1991;38(4–5):264–72. 1777808 10.1159/000114393

[7] Jarvis ED. Neural systems for vocal learning in birds and humans: a synopsis. J Ornithol [Internet]. 2007;148(1):35–44. Available from: http://eutils.ncbi.nlm.nih.gov/entrez/eutils/elink.fcgi?dbfrom=pubmed&id=19684872&retmode=ref&cmd=prlinks 10.1007/s10336-007-0243-0 19684872 PMC2726745

[8] Jarvis ED, Güntürkün O, Bruce L, Csillag A, Karten H, Kuenzel W, et al Opinion: Avian brains and a new understanding of vertebrate brain evolution. Nat Rev Neurosci [Internet]. 2011;6(2):151–9. 10.1038/nrn1606 PMC2507884 15685220

[9] Reiner A, Perkel DJ, Mello C V, Jarvis ED. Songbirds and the revised avian brain nomenclature. Ann N Y Acad Sci [Internet]. 2004;1016:77–108. Available from: http://eutils.ncbi.nlm.nih.gov/entrez/eutils/elink.fcgi?dbfrom=pubmed&id=15313771&retmode=ref&cmd=prlinks 10.1196/annals.1298.013 15313771 PMC2481519

[10] Hahnloser RHR, Kozhevnikov AA, Fee MS. An ultra-sparse code underlies the generation of neural sequences in a songbird. Nature [Internet]. 2002;419(6902):65–70. Available from: http://eutils.ncbi.nlm.nih.gov/entrez/eutils/elink.fcgi?dbfrom=pubmed&id=12214232&retmode=ref&cmd=prlinks 10.1038/nature00974 12214232

[11] Kozhevnikov AA, Fee MS. Singing-related activity of identified HVC neurons in the zebra finch. J Neurophysiol [Internet]. 2007;97(6):4271–83. Available from: http://eutils.ncbi.nlm.nih.gov/entrez/eutils/elink.fcgi?dbfrom=pubmed&id=17182906&retmode=ref&cmd=prlinks 10.1152/jn.00952.2006 17182906

[12] Long MA, Jin DZ, Fee MS. Support for a synaptic chain model of neuronal sequence generation. Nature [Internet]. 2010;468(7322):394–9. Available from: http://eutils.ncbi.nlm.nih.gov/entrez/eutils/elink.fcgi?dbfrom=pubmed&id=20972420&retmode=ref&cmd=prlinks 10.1038/nature09514 20972420 PMC2998755

[13] Leonardo A, Fee MS. Ensemble coding of vocal control in birdsong. J Neurosci [Internet]. 2005;25(3):652–61. Available from: http://eutils.ncbi.nlm.nih.gov/entrez/eutils/elink.fcgi?dbfrom=pubmed&id=15659602&retmode=ref&cmd=prlinks 10.1523/JNEUROSCI.3036-04.2005 15659602 PMC6725314

[14] Vicario DS, Nottebohm F. Organization of the zebra finch song control system: I. Representation of syringeal muscles in the hypoglossal nucleus. J Comp Neurol [Internet]. 1988/05/15 ed. 1988;271(3):346–54. Available from: http://www.ncbi.nlm.nih.gov/entrez/query.fcgi?cmd=Retrieve&db=PubMed&dopt=Citation&list_uids=3385013 10.1002/cne.902710305 3385013

[15] Vicario DS. Organization of the zebra finch song control system: functional organization of outputs from nucleus robustus archistriatalis. J Comp Neurol. 1991;309(4):486–94. 10.1002/cne.903090405 1655832

[16] Reinke H, Wild JM. Distribution and connections of inspiratory premotor neurons in the brainstem of the pigeon (Columba livia). J Comp Neurol. 1997;379:347–62. 9067829

[17] Reinke H, Wild JM. Identification and Connections of Inspiratory Premotor Neurons in Songbirds and Budgerigar. J Comp Neurol. 1998;391:147–63. 9518266

[18] Kubke MF, Yazaki-Sugiyama Y, Mooney R, Wild JM. Physiology of Neuronal Subtypes in the Respiratory-Vocal Integration Nucleus Retroamigualis of the Male Zebra Finch. J Neurophysiol [Internet]. 2005;94(4):2379–90. 10.1152/jn.00257.2005 15928060

[19] Dubbeldam J., den Boer-Visser A. The central mesencephalic grey in birds: nucleus intercollicularis and substantia grisea centralis. Brain Res Bull [Internet]. 2002 2 [cited 2015 Sep 29];57(3–4):349–52. Available from: http://www.sciencedirect.com/science/article/pii/S036192300100689X 11922987 10.1016/s0361-9230(01)00689-x

[20] Nottebohm F, Kelley DB, Paton JA. Connections of vocal control nuclei in the canary telencephalon. J Comp Neurol [Internet]. 1982/06/01 ed. 1982;207(4):344–57. Available from: http://www.ncbi.nlm.nih.gov/entrez/query.fcgi?cmd=Retrieve&db=PubMed&dopt=Citation&list_uids=7119147 10.1002/cne.902070406 7119147

[21] Striedter GF, Vu ET. Bilateral feedback projections to the forebrain in the premotor network for singing in zebra finches. J Neurobiol [Internet]. 1998 1;34(1):27–40. Available from: http://www.ncbi.nlm.nih.gov/pubmed/9469616 9469616

[22] Akutagawa E, Konishi M. New brain pathways found in the vocal control system of a songbird. J Comp Neurol. 2010;518(15):3086–100. 10.1002/cne.22383 20533361

[23] Ashmore RC, Wild JM, Schmidt MF. Brainstem and forebrain contributions to the generation of learned motor behaviors for song. J Neurosci [Internet]. 2005;25(37):8543–54. Available from: http://eutils.ncbi.nlm.nih.gov/entrez/eutils/elink.fcgi?dbfrom=pubmed&id=16162936&retmode=ref&cmd=prlinks 10.1523/JNEUROSCI.1668-05.2005 16162936 PMC6725679

[24] Ashmore RC, Renk JA, Schmidt MF. Bottom-up activation of the vocal motor forebrain by the respiratory brainstem. J Neurosci [Internet]. 2008;28(10):2613–23. Available from: http://eutils.ncbi.nlm.nih.gov/entrez/eutils/elink.fcgi?dbfrom=pubmed&id=18322104&retmode=ref&cmd=prlinks 10.1523/JNEUROSCI.4547-07.2008 18322104 PMC3263362

[25] Hamaguchi K, Tanaka M, Mooney R. A Distributed Recurrent Network Contributes to Temporally Precise Vocalizations. Neuron [Internet]. 2016 12 11;91(3):680–93. 10.1016/j.neuron.2016.06.019 27397518 PMC4975959

[26] Williams H, Vicario DS. Temporal patterning of song production: participation of nucleus uvaeformis of the thalamus. J Neurobiol [Internet]. 1993 7;24(7):903–12. Available from: http://www.ncbi.nlm.nih.gov/pubmed/8228968 10.1002/neu.480240704 8228968

[27] Coleman MJ, Vu ET. Recovery of impaired songs following unilateral but not bilateral lesions of nucleus uvaeformis of adult zebra finches. J Neurobiol. 2005;63(1):70–89. 10.1002/neu.20122 15685609

[28] Schmidt MF. Pattern of interhemispheric synchronization in HVc during singing correlates with key transitions in the song pattern. J Neurophysiol [Internet]. 2003;90(6):3931–49. Available from: http://eutils.ncbi.nlm.nih.gov/entrez/eutils/elink.fcgi?dbfrom=pubmed&id=12944542&retmode=ref&cmd=prlinks 10.1152/jn.00003.2003 12944542

[29] Gibb L, Gentner TQ, Abarbanel HDI. Brain Stem Feedback in a Computational Model of Birdsong Sequencing. J Neurophysiol [Internet]. 2009 9 24;102(3):1763–78. Available from: http://www.ncbi.nlm.nih.gov/pmc/articles/PMC2746794/ 10.1152/jn.91154.2008 19553477 PMC2746794

[30] Schmidt MF, Ashmore RC, Vu ET. Bilateral control and interhemispheric coordination in the avian song motor system. Ann N Y Acad Sci. 2004;1016:171–86. 10.1196/annals.1298.014 15313775

[31] Amador A, Perl YS, Mindlin GB, Margoliash D. Elemental gesture dynamics are encoded by song premotor cortical neurons. Nature. 2013 2;495(X):59–64. 23446354 10.1038/nature11967 PMC3878432

[32] Alonso RG, Trevisan M a, Amador A, Goller F, Mindlin GB. A circular model for song motor control in Serinus canaria. Front Comput Neurosci [Internet]. 2015 1 [cited 2016 Jan 30];9(4):41 Available from: http://www.pubmedcentral.nih.gov/articlerender.fcgi?artid=4387923&tool=pmcentrez&rendertype=abstract 10.3389/fncom.2015.00041 25904860 PMC4387923

[33] Fee MS, Kozhevnikov AA, Hahnloser RHR. Neural mechanisms of vocal sequence generation in the songbird. Ann N Y Acad Sci [Internet]. 2004;1016:153–70. Available from: http://eutils.ncbi.nlm.nih.gov/entrez/eutils/elink.fcgi?dbfrom=pubmed&id=15313774&retmode=ref&cmd=prlinks 10.1196/annals.1298.022 15313774

[34] Long MA, Fee MS. Using temperature to analyse temporal dynamics in the songbird motor pathway. Nature [Internet]. 2008;456(7219):189–94. Available from: http://eutils.ncbi.nlm.nih.gov/entrez/eutils/elink.fcgi?dbfrom=pubmed&id=19005546&retmode=ref&cmd=prlinks 10.1038/nature07448 19005546 PMC2723166

[35] Glaze CM, Troyer TW. Behavioral measurements of a temporally precise motor code for birdsong. J Neurosci. 2007;27(29):7631–9. 10.1523/JNEUROSCI.1065-07.2007 17634357 PMC6672882

[36] Hanuschkin A, Diesmann M, Morrison A. A reafferent and feed-forward model of song syntax generation in the Bengalese finch. J Comput Neurosci [Internet]. 2011 11 15;31(3):509–32. Available from: http://www.ncbi.nlm.nih.gov/pmc/articles/PMC3232349/ 10.1007/s10827-011-0318-z 21404048 PMC3232349

[37] Jin DZ, Ramazanoğlu FM, Seung HS. Intrinsic bursting enhances the robustness of a neural network model of sequence generation by avian brain area HVC. J Comput Neurosci. 2007;23(3):283–99. 10.1007/s10827-007-0032-z 17440800

[38] Li M, Greenside H. Stable propagation of a burst through a one-dimensional homogeneous excitatory chain model of songbird nucleus HVC. Phys Rev E Stat Nonlin Soft Matter Phys. 2006;74(1 Pt 1):11918. 10.1103/PhysRevE.74.011918 16907138

[39] Fee MS, Long MA. Neural Mechanisms underlying the generation of Birdsong: A Modular Sequential Behavior In: Birdsong, speech, and language: exploring the evolution of mind and brain. MIT press; 2013 p. 353–78.

[40] Andalman AS, Foerster JN, Fee MS. Control of vocal and respiratory patterns in birdsong: dissection of forebrain and brainstem mechanisms using temperature. PLoS One [Internet]. 2011;6(9):e25461 Available from: http://eutils.ncbi.nlm.nih.gov/entrez/eutils/elink.fcgi?dbfrom=pubmed&id=21980466&retmode=ref&cmd=prlinks 10.1371/journal.pone.0025461 21980466 PMC3182229

[41] Veit L, Aronov D, Fee MS. Learning to breathe and sing: development of respiratory-vocal coordination in young songbirds. J Neurophysiol [Internet]. 2011;106(4):1747–65. Available from: http://eutils.ncbi.nlm.nih.gov/entrez/eutils/elink.fcgi?dbfrom=pubmed&id=21697438&retmode=ref&cmd=prlinks 10.1152/jn.00247.2011 21697438 PMC3191841

[42] Lilliefors H. On the Kolmogorov–Smirnov test for the exponential distribution with mean unknown. J Am Stat Assoc. 1969;64:387–9.

[43] Aronov D, Andalman AS, Fee MS. A specialized forebrain circuit for vocal babbling in the juvenile songbird. Science [Internet]. 2008;320(5876):630–4. Available from: http://eutils.ncbi.nlm.nih.gov/entrez/eutils/elink.fcgi?dbfrom=pubmed&id=18451295&retmode=ref&cmd=prlinks 10.1126/science.1155140 18451295

[44] Aronov D, Veit L, Goldberg JH, Fee MS. Two distinct modes of forebrain circuit dynamics underlie temporal patterning in the vocalizations of young songbirds. J Neurosci [Internet]. 2011;31(45):16353–68. Available from: http://eutils.ncbi.nlm.nih.gov/entrez/eutils/elink.fcgi?dbfrom=pubmed&id=22072687&retmode=ref&cmd=prlinks 10.1523/JNEUROSCI.3009-11.2011 22072687 PMC3241969

[45] Saar S, Mitra PP. A technique for characterizing the development of rhythms in bird song. PLoS One. 2008;3(1):e1461 10.1371/journal.pone.0001461 18213370 PMC2180191

[46] Tchernichovski O, Mitra PP, Lints T, Nottebohm F. Dynamics of the vocal imitation process: how a zebra finch learns its song. Science [Internet]. 2001;291(5513):2564–9. Available from: http://eutils.ncbi.nlm.nih.gov/entrez/eutils/elink.fcgi?dbfrom=pubmed&id=11283361&retmode=ref&cmd=prlinks 10.1126/science.1058522 11283361

[47] Okubo TS, Mackevicius EL, Payne HL, Lynch GF, Fee MS. Growth and splitting of neural sequences in songbird vocal development. Nature [Internet]. 2015 12 17;528(7582):352–7. 10.1038/nature15741 26618871 PMC4957523

[48] Liu W, Gardner TJ, Nottebohm F. Juvenile zebra finches can use multiple strategies to learn the same song. Proc Natl Acad Sci U S A [Internet]. 2004;101(52):18177–82. Available from: http://eutils.ncbi.nlm.nih.gov/entrez/eutils/elink.fcgi?dbfrom=pubmed&id=15608063&retmode=ref&cmd=prlinks 10.1073/pnas.0408065101 15608063 PMC539774

[49] Tchernichovski O, Nottebohm F, Ho CE, Pesaran B, Mitra PP. A procedure for an automated measurement of song similarity. Anim Behav [Internet]. 2000/07/06 ed. 2000;59(6):1167–76. Available from: http://www.ncbi.nlm.nih.gov/entrez/query.fcgi?cmd=Retrieve&db=PubMed&dopt=Citation&list_uids=10877896 10.1006/anbe.1999.1416 10877896

[50] Boari S, Perl YS, Amador A, Margoliash D, Mindlin GB. Automatic reconstruction of physiological gestures used in a model of birdsong production. J Neurophysiol [Internet]. 2015 11 17;114(5):2912–22. Available from: http://jn.physiology.org/content/114/5/2912.abstract 10.1152/jn.00385.2015 26378204 PMC4737419

[51] Marler P. Birdsong: The acquisition of a learned motor skill. Trends Neurosci [Internet]. 1981;4:88–94. Available from: http://www.sciencedirect.com/science/article/pii/0166223681900291

[52] Picardo MA, Merel J, Katlowitz KA, Vallentin D, Okobi DE, Benezra SE, et al Population-Level Representation of a Temporal Sequence Underlying Song Production in the Zebra Finch. Neuron [Internet]. 2016 5 18;90(4):866–76. 10.1016/j.neuron.2016.02.016 27196976 PMC4941616

[53] Lynch G, Okubo TS, Hanuschkin A, Hahnloser RHR, Fee MS. Rhythmic continuous-time coding in the songbird analog of vocal motor cortex. Neuron. 2016;90(4):877–92. 10.1016/j.neuron.2016.04.021 27196977

[54] Mauk MD, Buonomano D V. THE NEURAL BASIS OF TEMPORAL PROCESSING. Annu Rev Neurosci [Internet]. 2004 6 24;27(1):307–40. 15217335 10.1146/annurev.neuro.27.070203.144247

[55] Amari S-I. Learning patterns and pattern sequences by self-organizing nets of threshold elements. Comput IEEE Trans. 1972;100(11):1197–206.

[56] Abeles M. Corticonics: Neural circuits of the cerebral cortex. Cambridge Univ. Press; 1991.

[57] Glaze CM, Troyer TW. Temporal structure in zebra finch song: implications for motor coding. J Neurosci. 2006;26(3):991–1005. 10.1523/JNEUROSCI.3387-05.2006 16421319 PMC6675377

[58] Cynx J. Experimental determination of a unit of song production in the zebra finch (Taeniopygia guttata). J Comp Psychol. 1990;104(1):3–10. 2354628 10.1037/0735-7036.104.1.3

[59] Ashmore RC, Bourjaily M, Schmidt MF. Hemispheric coordination is necessary for song production in adult birds: implications for a dual role for forebrain nuclei in vocal motor control. J Neurophysiol [Internet]. 2008;99(1):373–85. Available from: http://eutils.ncbi.nlm.nih.gov/entrez/eutils/elink.fcgi?dbfrom=pubmed&id=17977927&retmode=ref&cmd=prlinks 10.1152/jn.00830.2007 17977927

[60] Schmidt MF. Using both sides of your brain: the case for rapid interhemispheric switching. PLoS Biol [Internet]. 2008;6(10):e269 Available from: http://eutils.ncbi.nlm.nih.gov/entrez/eutils/elink.fcgi?dbfrom=pubmed&id=18959487&retmode=ref&cmd=prlinks 10.1371/journal.pbio.0060269 18959487 PMC2573940

[61] Otchy TM, Wolff SBE, Rhee JY, Pehlevan C, Kawai R, Kempf A, et al Acute off-target effects of neural circuit manipulations. Nature. 2015;528(7582):358–63. 10.1038/nature16442 26649821

[62] Goldin MA, Alonso LM, Alliende JA, Goller F, Mindlin GB. Temperature Induced Syllable Breaking Unveils Nonlinearly Interacting Timescales in Birdsong Motor Pathway. PLoS One [Internet]. 2013 6 20;8(6):e67814 10.1371/journal.pone.0067814 23818988 PMC3688611

[63] Sherman SM, Guillery RW. Functional organization of thalamocortical relays. J Neurophysiol [Internet]. 1996 9;76(3):1367–95. Available from: http://www.ncbi.nlm.nih.gov/pubmed/8890259 8890259 10.1152/jn.1996.76.3.1367

[64] Guillery R, Sherman S. Thalamic relay functions and their role in corticocortical communication: generalizations from the visual system. Neuron [Internet]. 2002 [cited 2014 Jun 23];33:163–75. Available from: http://www.sciencedirect.com/science/article/pii/S0896627301005827 11804565 10.1016/s0896-6273(01)00582-7

[65] Fee MS, Mitra PP, Kleinfeld D. Central versus peripheral determinants of patterned spike activity in rat vibrissa cortex during whisking. J Neurophysiol [Internet]. 1997;78(2):1144–9. Available from: http://eutils.ncbi.nlm.nih.gov/entrez/eutils/elink.fcgi?dbfrom=pubmed&id=9307141&retmode=ref&cmd=prlinks 9307141 10.1152/jn.1997.78.2.1144

[66] Hopkins CD. Stimulus filtering and electroreception: tuberous electroreceptors in three species of gymnotoid fish. J Comp Physiol. 1976;111(2):171–207.

[67] Guthrie BL, Porter JD, Sparks DL. Corollary discharge provides accurate eye position information to the oculomotor system. Science (80-). 1983;221(4616):1193–5. 6612334 10.1126/science.6612334

[68] Wurtz RH, Richmond BJ, Judge SJ. Vision during saccadic eye movements. III. Visual interactions in monkey superior colliculus. J Neurophysiol [Internet]. 1980 4 1;43(4):1168–81. Available from: http://jn.physiology.org/content/43/4/1168.abstract 6766998 10.1152/jn.1980.43.4.1168

[69] Wurtz RH, Sommer M a, Cavanaugh J. Drivers from the deep: the contribution of collicular input to thalamocortical processing. Prog Brain Res [Internet]. 2005 1 [cited 2014 Jun 20];149:207–25. Available from: http://www.ncbi.nlm.nih.gov/pubmed/16226586 10.1016/S0079-6123(05)49015-9 16226586

[70] Sommer MA, Wurtz RH. What the brain stem tells the frontal cortex. II. Role of the SC-MD-FEF pathway in corollary discharge. J Neurophysiol [Internet]. 2004;91(3):1403–23. Available from: http://eutils.ncbi.nlm.nih.gov/entrez/eutils/elink.fcgi?dbfrom=pubmed&id=14573557&retmode=ref&cmd=prlinks 10.1152/jn.00740.2003 14573557

[71] Sommer MA, Wurtz RH. What the brain stem tells the frontal cortex. I. Oculomotor signals sent from superior colliculus to frontal eye field via mediodorsal thalamus. J Neurophysiol [Internet]. 2004;91(3):1381–402. Available from: http://eutils.ncbi.nlm.nih.gov/entrez/eutils/elink.fcgi?dbfrom=pubmed&id=14573558&retmode=ref&cmd=prlinks 10.1152/jn.00738.2003 14573558

[72] Fee MS, Leonardo A. Miniature motorized microdrive and commutator system for chronic neural recording in small animals. J Neurosci Methods [Internet]. 2001;112(2):83–94. Available from: http://eutils.ncbi.nlm.nih.gov/entrez/eutils/elink.fcgi?dbfrom=pubmed&id=11716944&retmode=ref&cmd=prlinks 11716944 10.1016/s0165-0270(01)00426-5

[73] Okubo TS, Mackevicius EL, Fee MS. In Vivo Recording of Single-Unit Activity during Singing in Zebra Finches. Cold Spring Harb Protoc [Internet]. 2014 12 23;2014(12):1273–83. Available from: http://www.ncbi.nlm.nih.gov/pmc/articles/PMC4462520/ 10.1101/pdb.prot084624 25342072 PMC4462520

[74] Olveczky BP, Andalman AS, Fee MS. Vocal experimentation in the juvenile songbird requires a basal ganglia circuit. PLoS Biol [Internet]. 2005;3(5):902–9. Available from: http://eutils.ncbi.nlm.nih.gov/entrez/eutils/elink.fcgi?dbfrom=pubmed&id=15826219&retmode=ref&cmd=prlinks 10.1371/journal.pbio.0030153 PMC1069649 15826219

[75] Leonardo A. Experimental test of the birdsong error-correction model. Proc Natl Acad Sci U S A [Internet]. 2004 11 30;101(48):16935–40. Available from: http://www.ncbi.nlm.nih.gov/pmc/articles/PMC534752/ 10.1073/pnas.0407870101 15557558 PMC534752

[76] Perl YS, Arneodo EM, Amador A, Goller F, Mindlin GB. Reconstruction of physiological instructions from Zebra finch song. Phys Rev E [Internet]. 2011 11 16;84(5):51909. 10.1103/PhysRevE.84.051909 PMC3909473 22181446

[77] Mitra P, Bokil H. Observed brain dynamics. Oxford University Press; 2007.

