# Supplementary figures and images for: Rhythmic syllable-related activity in a songbird motor thalamic nucleus necessary for learned vocalizations
Source: PLoS One. 2017 Jun 15;12(6):e0169568. doi: 10.1371/journal.pone.0169568 (PMC5472270; doi:10.1371/journal.pone.0169568)

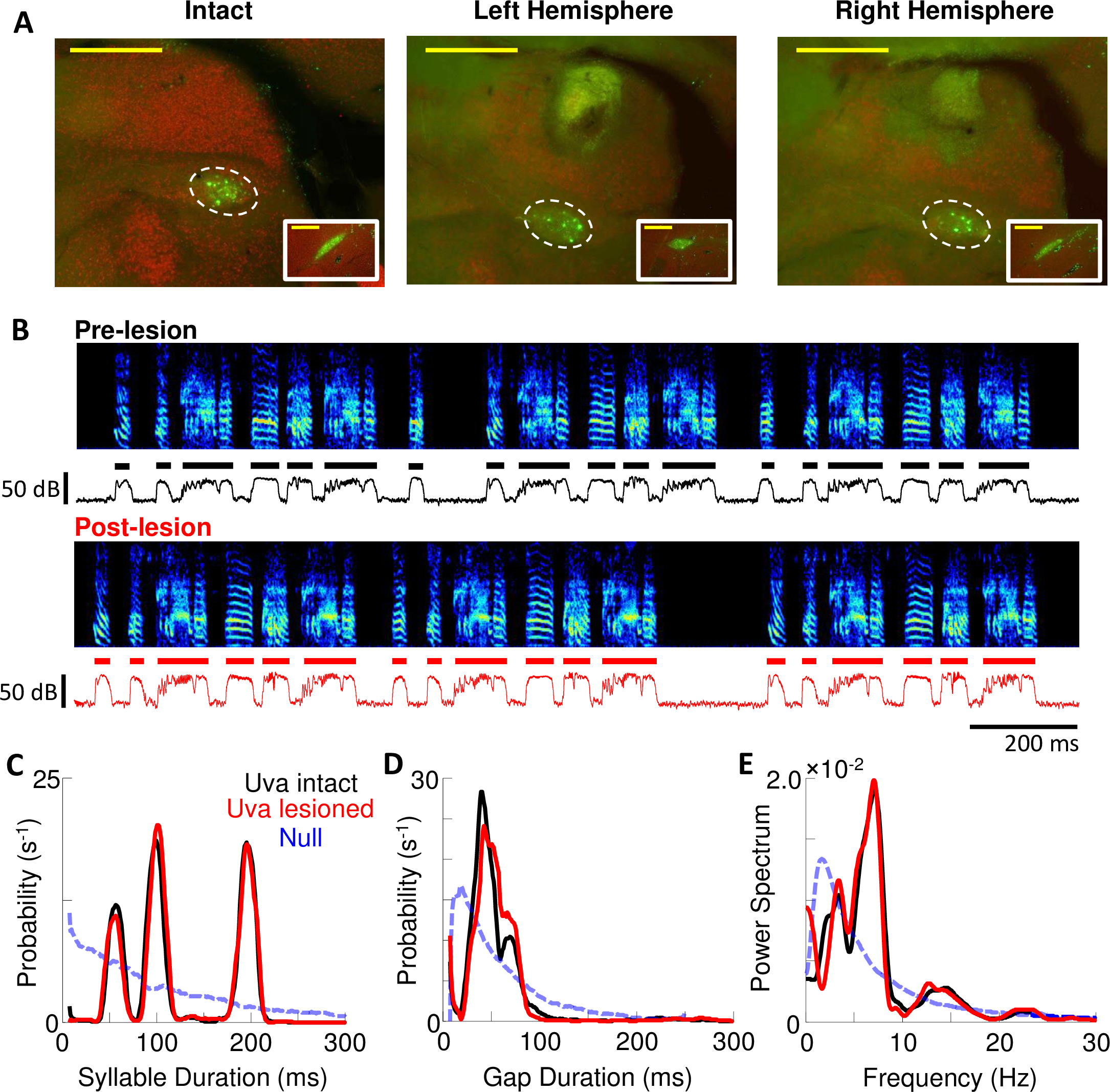

Supplement: S1 Fig — (A) (left) Retrograde tracers from HVC (dextran-conjugated Alexa Fluor 480) were used to distinguish Uva-HVC projectors from surrounding thalamic neurons (Neu-N, red) in an intact bird (yellow scale bar = 200μm). (middle, right) Absence of Neu-N stain reveals bilateral elimination of brain regions surrounding Uva. Dotted-line marks the border of Uva. (Inset)retrograde labeling from HVC in intact NIf demonstrate successful retrograde tracing (scale bar = 200μm) (B) (top) Prelesion song spectrogram of an adult bird (>90dph). Bottom trace is the song amplitude and the black segments indicate individual syllables. (Bottom) song spectrogram of the same bird taken from the first day of singing after bilateral control lesions. Note the song stereotypy in the duration of syllables and gaps between syllables, as well as the acoustic features of the song remains largely intact. (C) and (D) Distribution of syllable and gap durations, respectively, before (black trace) and after(red trace) bilateral control lesion. The null distribution for syllables and gaps is represented by an exponential or unimodal distribution, respectively (dashed blue trace). (E) normalized power spectra of the song amplitudes before and after lesion. Null power spectrum distribution (dotted blue) was generated from an exponential distribution of syllable durations and a unimodal distribution of gap durations. (TIF) [file pone.0169568.s002.tif]

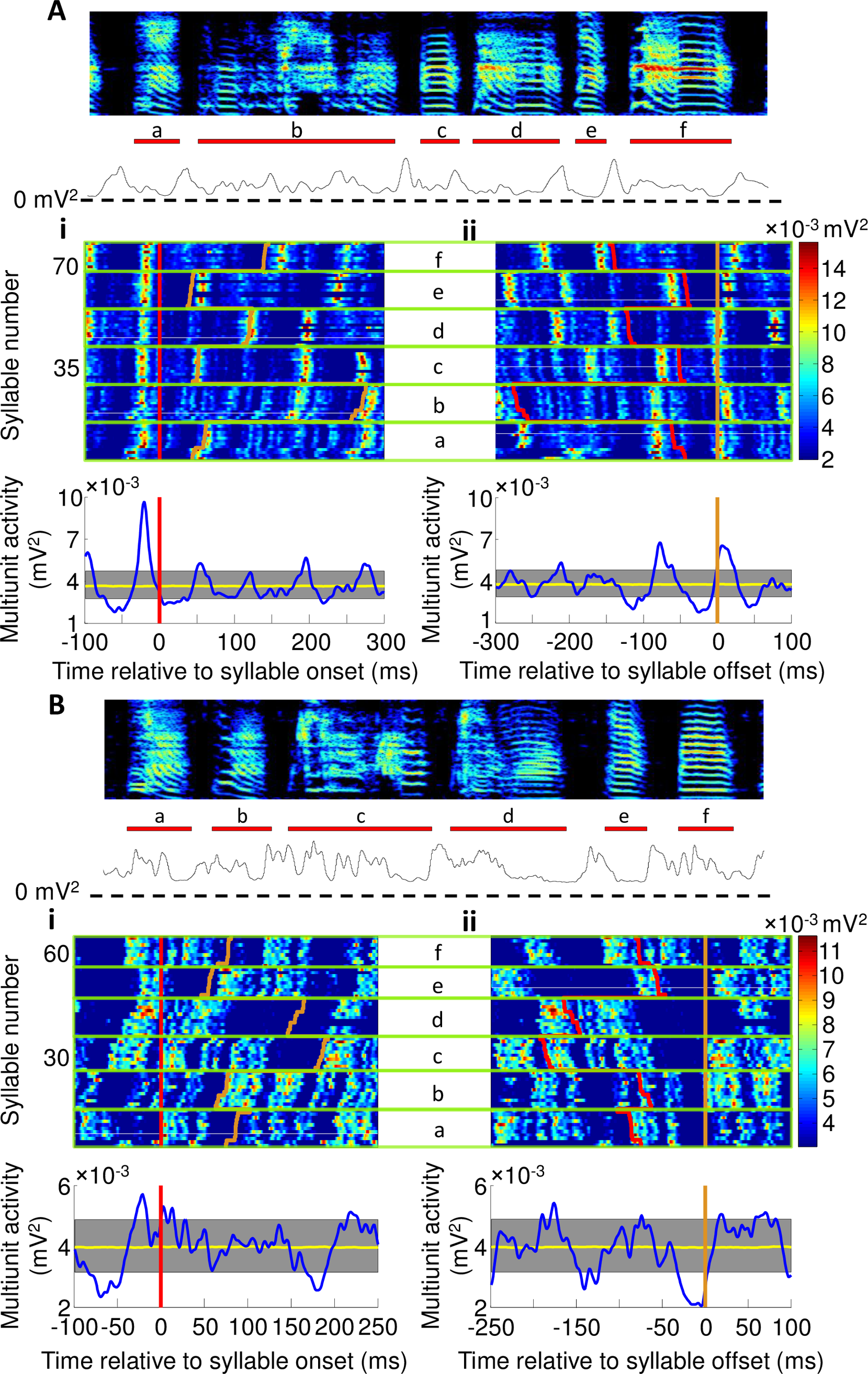

Supplement: S2 Fig — Above is a spectrogram of a single motif. Red bars represent the syllable lengths, with syllable labels below. (A) (i) Uva activity peaks prior to syllable onset. Raster(top) represents the power of neural activity during each syllable rendition. Red line marks syllable onset and orange line marks syllable offset. Syllables are grouped based on identity, arranged from longest to shortest syllable in descending order and then aligned to syllable onset. Individual syllables have been identified and labeled. Below is a syllable onset aligned multiunit trace averaged across all syllables. Also shown is the baseline activity during vocalization determined from random shuffling of multiunit activity (yellow; shading indicates 95% confidence interval for maxima and minima anywhere in this window). (ii) Uva activity dips prior to syllable offset. Heat raster (top) shows all syllables aligned to syllable offset. Average trace (below) shows a dip prior to syllable offset. (B) Data from an additional bird. (TIF) [file pone.0169568.s003.tif]

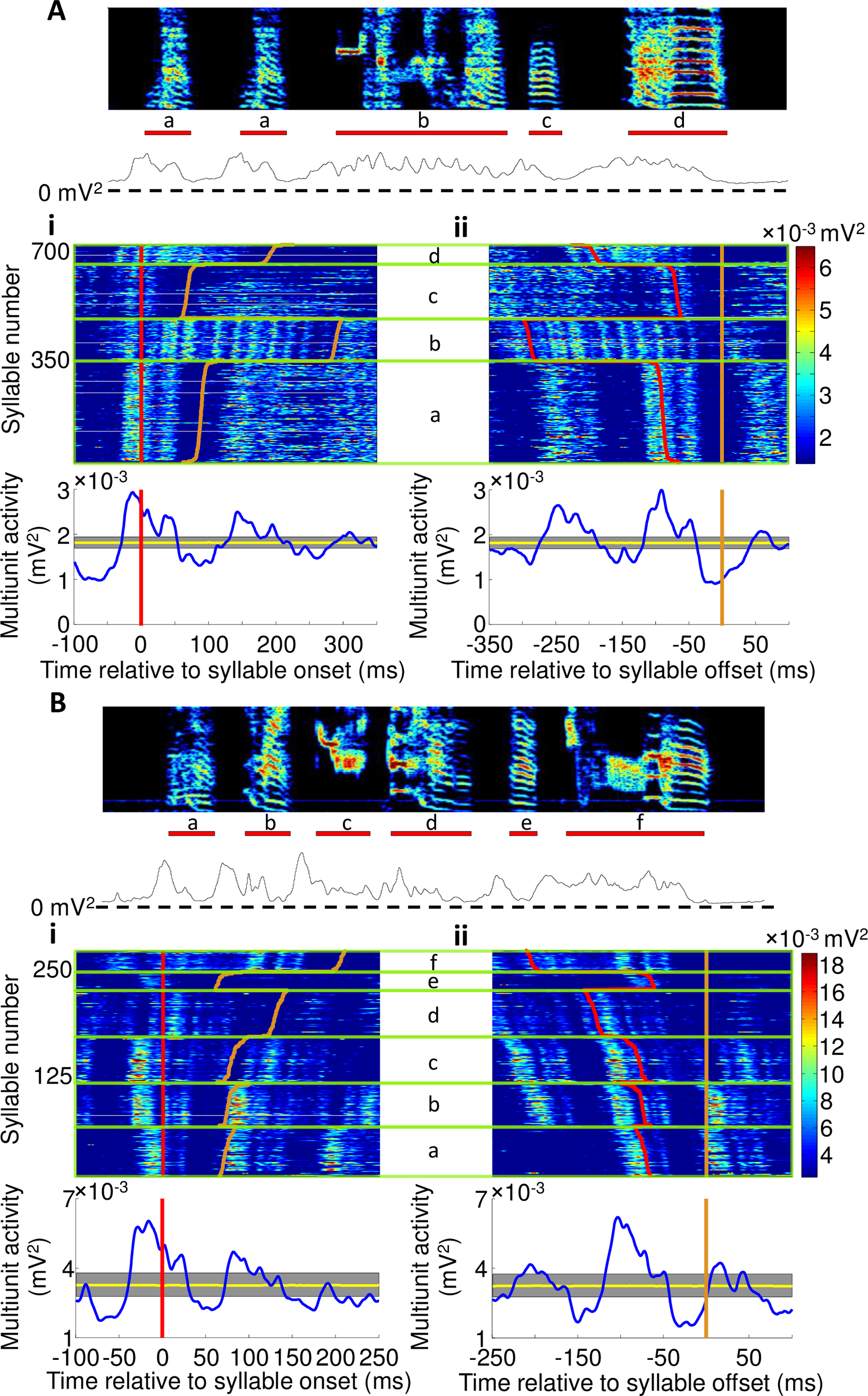

Supplement: S3 Fig — Above is a spectrogram of a single motif. Red bars represent the syllable lengths, with syllable labels below. (A) (i) Uva activity peaks prior to syllable onset. Raster(top) represents the power of neural activity during each syllable rendition. Red line marks syllable onset and orange line marks syllable offset. Syllables are grouped based on identity, arranged from longest to shortest syllable in descending order and then aligned to syllable onset. Individual syllables have been identified and labeled. Below is a syllable onset aligned multiunit trace averaged across all syllables. Also shown is the baseline activity during vocalization determined from random shuffling of multiunit activity (yellow; shading indicates 95% confidence interval for maxima and minima anywhere in this window). (ii) Uva activity dips prior to syllable offset. Heat raster (top) shows all syllables aligned to syllable offset. Average trace (below) shows a dip prior to syllable offset. (B) Data from an additional bird. (TIF) [file pone.0169568.s004.tif]

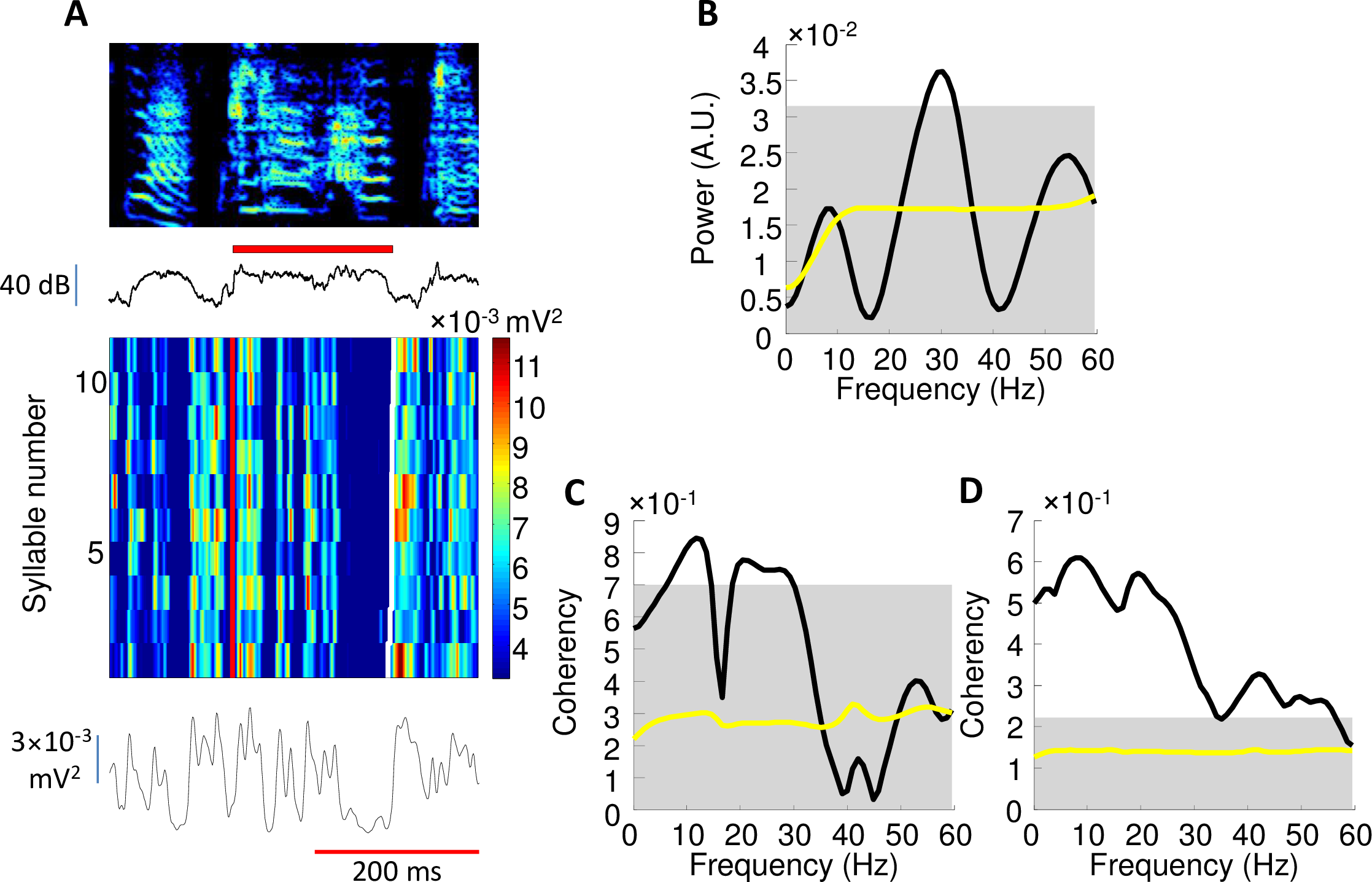

Supplement: S4 Fig — (A) In many long syllables (>150ms in length), we observed rapid oscillations in Uva activity. (B) In these long syllables, we consistently observed a peak in the power spectrum of Uva activity at frequencies in the gamma range. (C) Uva activity during these long syllables is significantly coherent with song amplitude across a large frequency range (1-55Hz) and (D) across all birds when compared to null distribution (yellow), calculated from randomly shuffled neural data (see Methods) (gray shading indicates 95% confidence interval for maxima anywhere in this window). (TIF) [file pone.0169568.s005.tif]

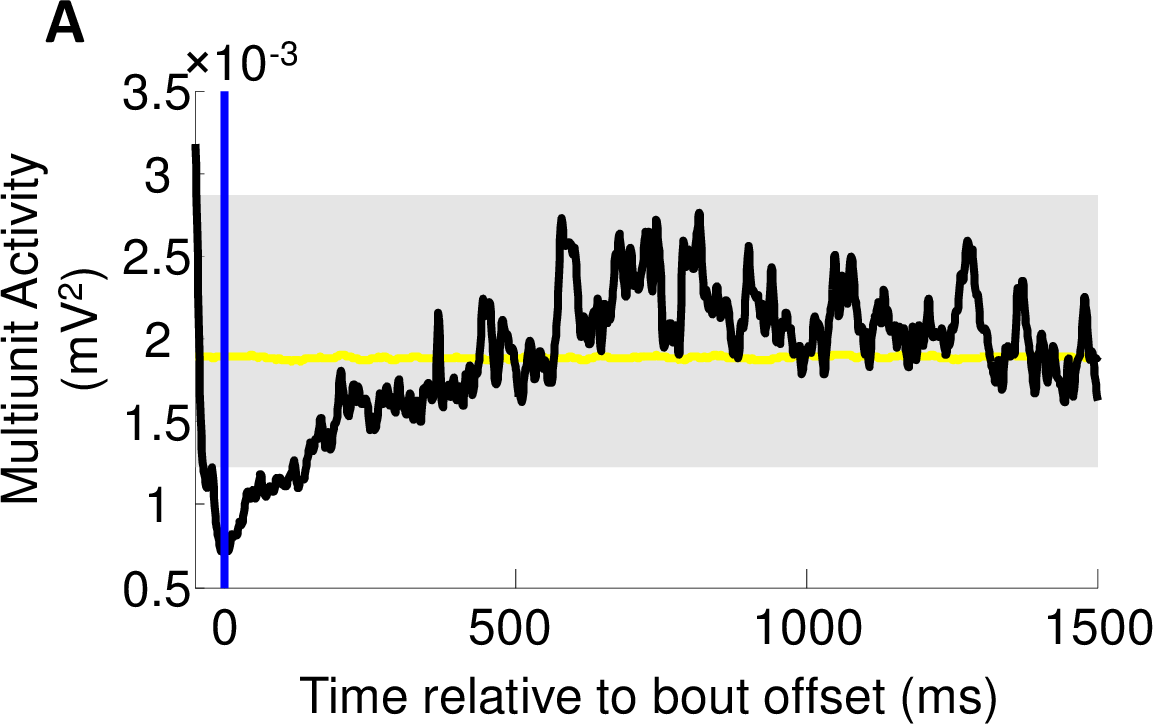

Supplement: S5 Fig — (A) A bout offset (blue) aligned multiunit trace averaged across all bouts (black). Also shown is the baseline activity during non-singing determined from random shuffling of multiunit activity (yellow; shading indicates 95% confidence interval for maxima and minima anywhere in this window). (TIF) [file pone.0169568.s006.tif]
